# Ionomers From Kraft Lignin for Renewable Energy Applications

**DOI:** 10.3389/fchem.2020.00690

**Published:** 2020-08-26

**Authors:** Seefat Farzin, Tyler J. Johnson, Shyambo Chatterjee, Ehsan Zamani, Shudipto K. Dishari

**Affiliations:** Department of Chemical and Biomolecular Engineering, University of Nebraska-Lincoln, Lincoln, NE, United States

**Keywords:** ionomer, thin films, Nafion, kraft lignin, proton conduction, energy

## Abstract

Converting industrial/agricultural lignin-rich wastes to efficient, cost-effective materials for electrochemical devices (e.g., fuel cells) can aid in both bio- and energy economy. A major limitation of fuel cells is the weak ion conductivity within the ~2–30-nm thick, ion-conducting polymer (ionomer)-based catalyst-binder layer over electrodes. Here, we strategically sulfonated kraft lignin (a by-product of pulp and paper industries) to design ionomers with varied ion exchange capacities (IECs) (LS x; x = IEC) that can potentially overcome this interfacial ion conduction limitation. We measured the ion conductivity, water uptake, ionic domain characteristics, density, and predicted the water mobility/stiffness of Nafion, LS 1.6, and LS 3.1 in submicron-thick hydrated films. LS 1.6 showed ion conductivity an order of magnitude higher than Nafion and LS 3.1 in films with similar thickness. The ion conductivity of these films was not correlated to their water uptake and IECs. Within the three-dimensional, less dense, branched architecture of LS 1.6 macromolecules, the –SO_3_H and –OH groups are in close proximity, which likely facilitated the formation of larger ionic domains having highly mobile water molecules. As compared to LS 1.6, LS 3.1 showed a higher glass transition temperature and film stiffness at dry state, which sustained during humidification. On the contrary, Nafion stiffened significantly upon humidification. The smaller ionic cluster within stiff LS 3.1 and Nafion films thus led to ion conductivity lower than LS 1.6. Since LS x ionomers (unlike commercial lignosulfonate) are not water soluble, they are suitable for low-temperature, water-mediated ion conduction in submicron-thick films.

## Introduction

For environmental, economic, and societal growth, we need to attain both sustainable energy- and bioeconomy. The Department of Energy's Biotechnologies Office states that to achieve sustainable bioeconomy, we need to strategically design and utilize bio-based products from non-food waste sources (Strategic Plan for a Thriving and Sustainable Bioeconomy, 2016). Lignin is the second most abundant polymer in nature (next to cellulose) and accounts for 15–40 mass% of the plant cell walls (Aro and Fatehi, 2017). Besides, lignocellulosic biorefineries, and pulp and paper industries produce lignin-rich waste (>70 million ton/year) (Aro and Fatehi, 2017), only 1–2% of which is utilized to produce value-added chemicals (Aro and Fatehi, 2017), and the rest is usually combusted to generate heat (Aro and Fatehi, 2017). Some of the lignin valorization efforts have yielded products, like, concrete additives (Danner et al., 2015), plasticizers (Kalliola et al., 2015; Naseem et al., 2016), stabilizing agents (Cerrutti et al., 2012), dispersing agents (Konduri et al., 2015; Kai et al., 2016), corrosion inhibitors (Abu-dalo et al., 2013), thermoplastics (Lange et al., 2013), fuel (Che et al., 2018), aromatic diols (Che et al., 2018) (after delignification), block copolymers (Holmberg et al., 2014a,b) (from monolignols after breaking lignin), and more (Lange et al., 2013; Norgren and Edlund, 2014). To best utilize the abundant natural resources and make bioprocessing industries more economically viable, we critically need more unique lignin valorization efforts to design products with commercial and technological importance for sectors, like energy.

On the other hand, to achieve energy sustainability, we need low-cost, eco-friendly materials that can also overcome the technical barriers of energy conversion and storage devices (fuel cells, electrolyzers, batteries). A major technical challenge of the hydrogen fuel cell, one of the most promising energy conversion devices, is the ion conduction limitations (Astill et al., 2009; Peron et al., 2011; Holdcroft, 2013; Modestino et al., 2013; Kusoglu and Weber, 2017) at the interface of ion-conducting polymer (ionomer) and catalyst particles on electrodes. The current state-of-the-art ionomer Nafion conducts protons very efficiently in bulk membrane separator [~25–50-μm thick (Choi et al., 2005; Xu et al., 2011), used in between two electrodes to conduct protons from anode to cathode]. But when the same ionomer is coated over a substrate as a submicron-thick film (Dishari and Hickner, 2013; Holdcroft, 2013; Modestino et al., 2013; Dishari, 2014; Kusoglu and Weber, 2017; Farzin et al., 2019) or over catalyst particles on cathodes as a ~2–30-nm thick catalyst-binder (Astill et al., 2009; Peron et al., 2011; Holdcroft, 2013), it shows significantly lower proton conductivity. The confinement effect (Richter and Kruteva, 2019) and interfacial interactions (Keddie et al., 1994) between water, polymer, and substrate start to dominate as the film thickness approaches a few multiples of the radius of gyration (*R*_*g*_) of polymer chains (Innis-samson and Sakurai, 2011), impacting glass transition temperature (Keddie et al., 1994; Forrest and Dalnoki-veress, 2001; Roth and Dutcher, 2005), polymer chain diffusion coefficient/mobility (Frank and Gast, 1996), proton conductivity (Modestino et al., 2013), and many other properties (Campo, 2008; Smith et al., 2009). Moreover, Nafion is very expensive ($500/kg, according to the 2018 cost projection report of the Department of Energy's Fuel Cell Technologies Office) (James, 2018) and not eco-friendly because it is fluorocarbon based.

There have been significant efforts to design low cost, but efficiently proton-conducting, hydrocarbon-based ionomers to substitute fluorocarbon-based ionomers. Many of these ionomers exhibited proton conductivity comparable to or slightly higher than Nafion in bulk membrane format (Kreuer, 2001; Lufrano et al., 2001; Hickner et al., 2004; Miyatake et al., 2012; Chang et al., 2013, 2015; Li and Guiver, 2014; Lee et al., 2015; Li et al., 2015; Miyake et al., 2017) but they could not exceed Nafion considerable in sub-micron thick films. In general, poly(aromatic)-type hydrocarbon-based ionomers designed so far have some key attributes, which are distinctly different from fluorocarbon-based ionomers (Kreuer, 2001; Seung et al., 2004; Peron et al., 2011; Holdcroft, 2013), such as less acidic sulfonic acid groups at the side chain (pK_a_ between −1 and −2) (Kreuer, 2001; Peron et al., 2011) as compared to perfluorosulfonic acid (pK_a_ ~ −6) of Nafion) (Kreuer, 2001; Seung et al., 2004), less flexible backbone, less-pronounced hydrophilic-hydrophobic phase separation (Kreuer, 2001; Seung et al., 2004), low protonic mobility (Kreuer, 2001), and narrow ion-conducting channels (Kreuer, 2001) with dead-ends/pockets and high tortuosity (Kreuer, 2001). None of these features are favorable for proton conduction, especially for submicron-thick ionomer films (Astill et al., 2009; Peron et al., 2011; Holdcroft, 2013; Kusoglu and Weber, 2017; Karan, 2019). Therefore, just like perfluorinated ionomers, the reported polyaromatic ionomers experienced a drastic decrease in proton conductivity (about two orders of magnitude) (Ma et al., 2003; Peron et al., 2011) in catalyst layers from that in corresponding bulk membranes. The limited reports on polyaromatic ionomer-based catalyst layers suggested lower proton conductivity or higher binder-phase diffusion limitations as compared to Nafion (Ma et al., 2003; Sambandam and Ramani, 2010; Peron et al., 2011). Also, proton conductivity (Ma et al., 2003; Peron et al., 2011), and O_2_ permeability (Sambandam and Ramani, 2010) of a reaction layer (mimicking ionomer-based catalyst layer) made of sulfonated poly(ether sulfone) was significantly lower than a nafion-based one. These transport resistances are detrimental for oxygen reduction reaction kinetics and have hindered the transition from fluorocarbon to hydrocarbon-based catalyst binders. We, therefore, need new formulations of ionomers that are environment-friendly (fluorine-free), cost-effective, and at the same time, can conduct protons efficiently, especially under thin-film confinement.

Lignin has multiple structural features and characteristics, which makes it an ideal precursor of ionomer. Of the plant cell wall polymers, cellulose has been widely explored to make green electron (Zhu et al., 2016) or ion-conducting materials (Seo et al., 2009; Bayer et al., 2016; Zhu et al., 2016; Vilela et al., 2019). However, lignin has been underutilized as an ionomer and majorly used to synthesize porous carbon (graphene, carbon fiber) (Kontturi, 1988; Naseem et al., 2016) or charge storage materials (Kontturi, 1988) [requiring oxidation of phenol groups of lignin to quinone (Furman and Lonsky, 1988; Milczarek and Inganas, 2012; Milczarek and Nowicki, 2013; Thakur et al., 2014; Zhu et al., 2016) or mixing with quinone (Furman and Lonsky, 1988)]. Lignin is a three-dimensional (3D) amorphous, aromatic polymer functionalized with polar ether linkages (-O-) and hydroxyl (–OH) groups. These –OH groups are parts of p-coumaryl, coniferyl, and sinapyl alcohols, the three types of monolignols constituting lignin (Kim et al., 2011). The monolignols are connected with each other via interunit linkages (β-O-4, β-β, β-5, β-1, 5-5, and 4-O-5), giving the ultimate 3D, branched macromolecular structure and mechanical integrity (Kim et al., 2011). If ion-conducting functionalities, such as sulfonic acid (–SO_3_H) groups are covalently attached to the lignin structure, the neutral lignin polymer can act like an ionomer. The polar –OH groups and ether (-O-) linkages, alongside –SO_3_H groups, can attract water molecules inside the polymer network, form additional hydrogen-bonded (Grunwald and Puar, 1967; Nagamani et al., 2011, 2012; Ye et al., 2019) proton conduction pathways and facilitate water-mediated proton conduction within lignin sulfonate-based ionomeric materials. To date, a few works have been reported where neutral lignin (Uddin et al., 2017; Ye et al., 2019) or water-soluble, polyelectrolyte-like lignosulfonate (Zhang et al., 2006; Gonggo et al., 2012) were blended with a neutral polymer matrix material (polystyrene or polysulfone) to make composite membranes for methanol fuel cells and redox flow batteries. However, these demonstrations were limited to thick, bulk membranes only (not to submicron-thick films). Also, the lignosulfonates used in these reports (Zhang et al., 2006; Gonggo et al., 2012) and commercial lignosulfonates (Aro and Fatehi, 2017; Inwood et al., 2018) are highly water soluble that may lead to dissolution/degradation of ionomer membranes or thin films. The high water solubility of commercial lignosulfonate may originate from a high degree of sulfonation (Lange et al., 2013) (during the sulfite pulping process) and a low degree of cross-linking. Thus, for practical applicability in hydration-mediated ion conduction, we need to limit the water solubility of lignin sulfonate.

**Figure 1:**
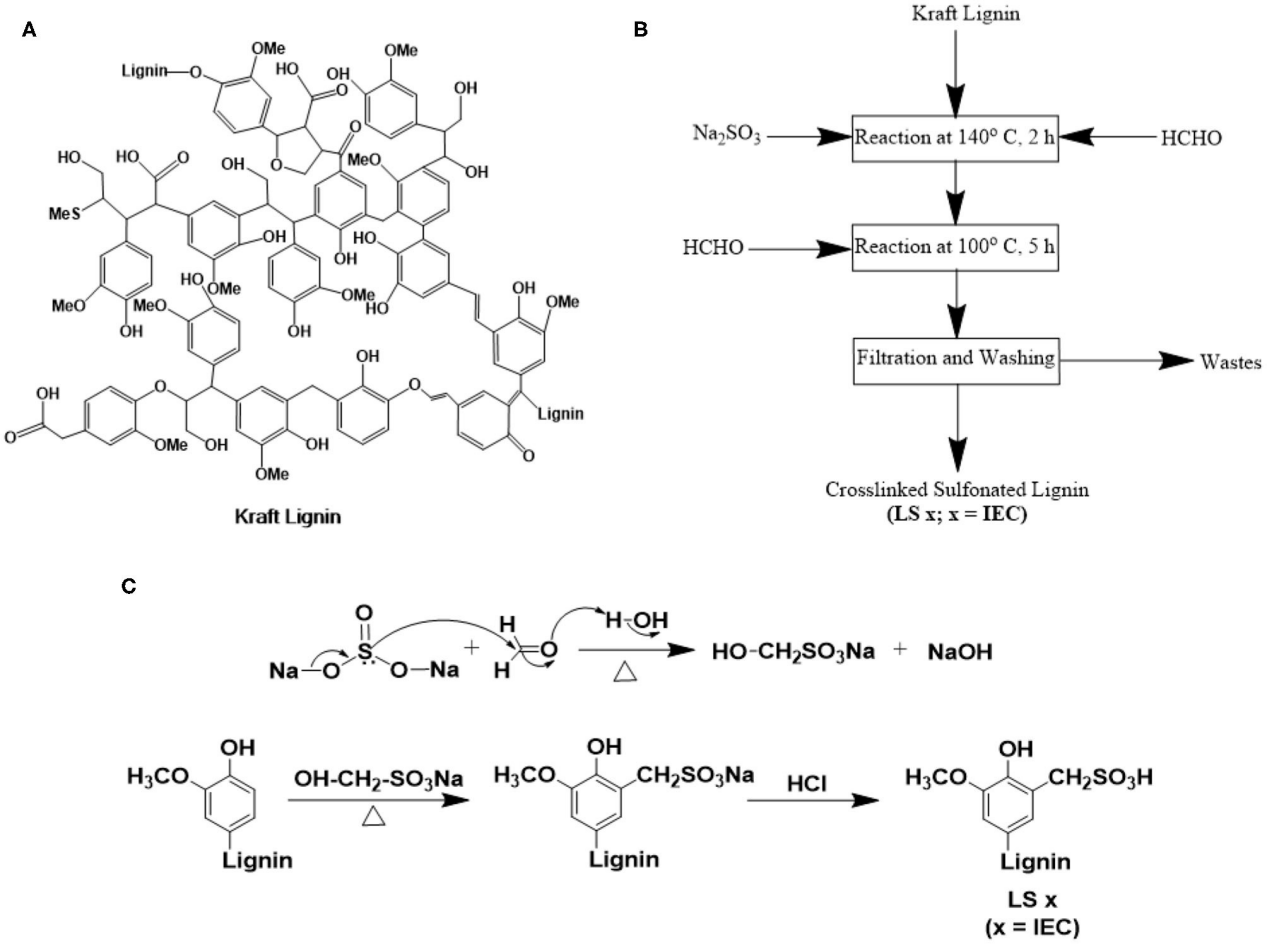
**(A)** Chemical structure of kraft lignin (Lange et al., 2013); **(B)** step-by-step procedure; and **(C)** reaction mechanism of sulfonation of kraft lignin to yield lignin sulfonate ionomers (LS x, where x = IEC).

As our first set of efforts along this direction, here we report kraft lignin-based synthesis of ionomers. We explored properties of these ionomers in submicron-thick films to demonstrate the excellent ion conduction properties of lignin-based ionomers under thin-film confinement. Kraft lignin (Figure 1A) is generally precipitated by acidification (using CO_2_ and/or H_2_SO_4_) of black liquor of kraft pulping (Lange et al., 2013; Thakur et al., 2014; Carvajal et al., 2016; Upton and Kasko, 2016; Aro and Fatehi, 2017) and is made commercially available as a water-insoluble (at low to neutral pH) (Thakur et al., 2014), neutral polymer [0.23–3 wt% sulfur, but no sulfonated content (Upton and Kasko, 2016; Aro and Fatehi, 2017)]. We sulfomethylated and subsequently cross-linked this commercial kraft lignin (Aro and Fatehi, 2017) to yield sulfonated lignin ionomers, LS x, where x is the ion exchange capacity of the ionomer (Figures 1B,C). By tuning the ratio of reactants (Aro and Fatehi, 2017), LS x ionomers with controlled ion exchange capacities (IECs) were achieved. Controlled sulfonation and subsequent cross-linking limited the water solubility of the LS x ionomers and fulfilled a critical requirement of practical ionomers for many forms of fuel cells, redox flow batteries, and electrolyzers. In order to understand and explain the route to the higher thin-film proton conductivity of LS x ionomers over Nafion, we measured the water uptake, density, ionic domain characteristics, and water mobility/stiffness (qualitative) of the same films as a function of relative humidity (% RH). While further investigations on mechanical, chemical, and thermal stabilities are needed for this new range of ionomers, this work provides valuable insights into the proton conduction and morphological behavior of LS x ionomers under thin-film confinement. This may guide future designs of lignin-based ionomers.

## Experimental Section

### Materials

Kraft lignin [isolated from Norway Spruce, M_n_ ~ 8,564 g/mol, M_w_ ~ 21,795 g/mol, polydispersity index (PDI) ~ 2.54], 20 wt% Nafion solution (EW ~ 1,100, IEC ~ 0.909 meq/g), and rotor probe [9-(2-carboxy-2-cyanovinyl) julolidine] (CCVJ) were purchased from MilliporeSigma (St. Louis, MO). Sulfonation was done using the same lot of kraft lignin to maintain the original composition of lignin consistent for all LS x ionomers. To compare with the LS x ionomers (water insoluble) prepared in this work, water-soluble sodium lignosulfonate (LScom) was also purchased (Santa Cruz Biotechnology, Dallas, TX) and studied. Silicon wafers coated with native oxide of silicon (n-SiO_2_ wafers) were purchased from Wafer Pro (San Jose, CA). n-SiO_2_-supported gold interdigitated electrode (IDE) arrays (Revtek, Inc., Torrance, CA) and n-SiO_2_ coated 5 MHz Au crystals (Inficon, Syracuse, NY) were purchased for impedance and water uptake measurements of ionomer films, respectively. All the solvents, reagents, and filter papers (Whatman, grade 1) were purchased from Fisher Scientific (Hampton, NH).

### Methods

#### Synthesis of Lignin Sulfonate (LS x) and Fractionation

The sulfonation of kraft lignin was carried out as described previously (Aro and Fatehi, 2017). Briefly, 1 g of kraft lignin was dissolved in 3 mL of formaldehyde (HCHO) to which anhydrous sodium sulfite (Na_2_SO_3_) was added and stirred for 2 h at 140°C to produce sulfomethylated kraft lignin. The ratio of lignin to Na_2_SO_3_ was maintained as 1:0.5, and 1:0.8 to obtain LS 1.6 and LS 3.1 ionomers, respectively. After 2 h, 2 mL of HCHO was added further to initiate the cross-linking reaction. The cross-linking took place for another 5 h at 100°C. In the end, a few drops (~200–300 μL) of 1 M HCl were added to the reaction mixture to convert –CH_2_SO_3_Na to –CH_2_SO_3_H groups. Once the reaction was complete, the reaction mixture was washed thrice with water (to remove water-soluble fractions of LS x), filtered using filter paper, and finally dried to yield LS x ionomers as brown solids. The yields of dried LS x were calculated to be ~69% for both IECs (1.6, 3.1). The experimentally measured molecular weights and PDIs were: LS 1.6: M_n_ ~ 11,000, M_w_ ~ 28,000, PDI ~ 2.54; and LS 3.1: M_n_ ~ 13,000, M_w_ ~ 33,427, PDI ~ 2.57. This PDI was significantly lower than that of commercial lignosulfonate from Norway Spruce (PDI ~ 4–9) (Lange et al., 2013).

The water-insoluble, dry powder of LS x was then mixed with an acetone-water (3:1) mixture, vortexed for 5 min, and ultrasonicated for 30 min. Even after ultrasonication, some of the LS x (~ <30%) did not dissolve in the acetone-water solvent system, which could be due to the higher degree of cross-linking. By centrifugation (8,500 rpm), the acetone-water soluble fraction of LS x was thus separated from undissolved fraction. This protocol was consistently followed for any further sample preparation.

#### IEC Determination

Typically, IECs of ionomers are calculated by soaking the dry ionomer in a 1 M NaCl solution to exchange the protons (H^+^) with Na^+^ ions. The H^+^ ions, transferred into the aqueous solution (Smitha et al., 2003; He and Frank, 2014) was then titrated with 0.01 N NaOH. Phenolphthalein was used during the titration as a pH indicator. Since LS x ionomers were brown powder, it was difficult to monitor the pH change during the titration. Therefore, as an alternate approach, we mixed LS x (the fraction soluble in 3:1 acetone: water) with Nafion (LS x:Nafion = 1:9 w/w) and made composite membranes, soaked in NaCl solution (for ion exchange and transfer of H^+^ from membrane to aqueous solution), and then titrated as mentioned earlier. Equation (1) was used to calculate the IECs of the composite membranes:

(1) $$\begin{array}{l} IEC_{composite}\left(meq/g\right)\\ =\frac{Normality\;of\;NaOH\times Volume\;of\;NaOH\;required\;\left(mL\right)}{Weight\;of\;dried\;polymer\;\left(g\right)} \end{array}$$

Equation (2) (Lavorgna, 2009) was then used to calculate the IECs of LS x present in the composite membranes:

(2) $$\begin{array}{l} {\left(IEC\right)}_{composite}\left(meq/g\right)={\left(wt\;fraction\right)}_{Nafion}\times IEC_{Nafion}\\ \;+{\left(wt\;fraction\right)}_{LS\;x}\times IEC_{LS\;x} \end{array}$$

We repeated this process in three replicates for each batch of LS x to confirm the IEC, and the values varied within ±2% of the one reported here. The sulfonation of kraft lignin through sulfomethylation reactions was also confirmed by performing the elemental analysis (described next).

#### Elemental Analysis

Elemental analysis of kraft lignin and LS x were carried out to determine the elemental composition by sending the samples to Galbraith Laboratories (Knoxville, TN). The samples were dried at 80°C for 6 h before the measurements. The wt% of carbon (C), hydrogen (H), and nitrogen (N) were measured using PerkinElmer 2400 Series II CHNS/O analyzer; while the Thermo Finnigan FlashEA^TM^ 1112 elemental analyzer and LECO SC-632 carbon/sulfur determinator were used to determine the wt% of oxygen (O), and sulfur (S), respectively.

#### Thermogravimetric Analysis (TGA)

TGA was carried out to determine the thermal stability and decomposition temperature of kraft lignin and LS x using TGA 209 F1 Libra (resolution: 0.1 μg) operating under a nitrogen environment. The scans were done from room temperature (25°C) to 600°C with a heating rate of 5°C/min.

#### Glass Transition Temperature (T_g_) Measurement

To determine the *T*_*g*_ of powder LS x ionomers (dried under vacuum at 100°C, overnight), differential scanning calorimetry measurements were carried out using DSC 204F1 Phoenix under nitrogen environment from room temperature 22°C to 250°C, with 5 and 10°C/min as heating and cooling rates, respectively.

#### Thin-Film Preparation

We started with the 1–10 wt% dry LS x powder and followed the protocol mentioned in the earlier section to prepare LS x ionomer solution dissolved in a 3:1 acetone-water mixture. This solution was then used to make LS x ionomer films with thickness ~20–200 nm. Also, 20 wt% Nafion stock solution was diluted with ethanol to yield 0.5–5 wt% dispersion for making Nafion films with similar thickness. n-SiO_2_ wafers were used as substrates for making ionomer films unless otherwise stated. The wafers were cut into small pieces (2 × 2.5 cm) and cleaned using the following steps: (1) dust particles were removed from substrate surface using compressed air, (2) the substrate was then rinsed with acetone and ethanol, dried with compressed air, and finally (3) treated using a UV-ozone cleaner for 20 min. The ionomer films were then spin-coated using EC 101 spin coater (Headway Research, Inc., Garland, TX) for 1.5 min. The spinning speed was varied in the range of 1,000–7,000 rpm to obtain films with different thicknesses. The spun films were placed inside a vacuum oven [VWR (Model # 1415 M), Radnor, PA], dried at 42°C for 3 h, annealed at 100°C for 7 h, and cooled down to room temperature for 12 h under vacuum. The films were then placed inside an appropriate humidity chamber and exposed to air with varied RHs for different measurements.

#### Thickness Measurement

The thickness of annealed films was measured at ambient condition (~20–30% RH) using variable angle (65–75°) spectroscopic ellipsometry (α-SE^TM^, J.A. Woollam Co., Inc., Lincoln, NE) with a spectral wavelength range of 381–893 nm. The thickness of the n-SiO_2_ on a bare silicon wafer (~1.77 nm) was used as a reference for ellipsometric modeling. The Cauchy model was used to obtain the thickness of all the ionomer films.

#### Ion Conductivity Measurement

The impedance of ionomer films was measured using a Solartron 1260a Impedance/Gain-Phase analyzer (Solartron Analytical, Leicester, England), which was connected to a probe station with four gold probes, a Peltier temperature stage, a temperature controller, and an environmental chamber (Nextron, Busan, South Korea). The thin-film impedance and ion conductivity were measured using a 2-microprobe-based technique as used by others (Modestino et al., 2013). Here, gold IDEs, fabricated on a silicon wafer (110) with a thermally grown SiO_2_ as an electrically insulating layer, were used as substrate. Each IDE had 110 gold teeth. Each tooth was 10-μm wide. The teeth were spaced 100 μm apart, with an overlapping length of 8 mm. Ionomer films (~20–250 nm thick) were spin-coated on these IDEs and annealed following the procedure mentioned in the “Thin-Film Preparation” section.

After annealing, the parts of the films covering the contact pads were removed using a small brush wetted with the same solvent as used to spin-coat the selected ionomers. This process was repeated as needed to ensure the contact pads were as clean as possible. The contact pads were allowed to dry before the impedance measurement. Then the film-coated IDEs were quickly placed on a temperature stage (set at 22°C) inside the environmental chamber, and the gold tip probes were carefully positioned onto the IDE contact pads. The environmental chamber was maintained at 90% RH by bubbling a stream of dry supply air [0.5 SCFH (~235 ccm)] through a bottle containing saturated Na_2_SO_4_ solution (placed in a water bath at 23°C) and passing the humid air through the environmental chamber. A humidity sensor was placed at the outlet of the environmental chamber to monitor and ensure that the target RH was attained and maintained inside the chamber.

After the sample was placed inside the chamber, its impedance was measured periodically in the range of 1–10 kHz to monitor the equilibration of the sample. Once equilibrated at 90% RH, the impedance response of the humidified ionomer film was collected in the range of 10 MHz−1 Hz at 100 mV AC potential. The impedance data were then fitted to an appropriate equivalent circuit model (Figure S3) using ZView software (Scribner Associates, Southern Pines, NC), which gave the value of film resistance (*R*_*f*_). The details of the rationale of choosing the appropriate components for the equivalent circuit model and the corresponding fits are presented in the supporting information (Figure S4 and Table S2). The film resistance (*R*_*f*_), film thickness (*t*), and the specifications of the IDEs [spacing between teeth of IDE electrodes (*d*), length of each teeth (*l*), number of teeth (*N*)] were then used in the Equation (3) to calculate the values of ion conductivity (*k*_*f*_):

(3) $$\begin{array}{l} k_{f}=\frac{1}{R_{f}}\frac{d}{l\left(N-1\right)t} \end{array}$$

Impedance measurements were done using two different IDE designs where IDE teeth spacing (100 and 40 μm) and teeth width (10 and 8 μm) were varied. In both cases, the obtained film resistance values were similar (within 10–20% variation between measurements on two IDEs). Therefore, for all the measurements reported here, IDEs with teeth spacing of 100 μm and width of 10 μm were used.

#### Water Uptake Measurement

Water uptake of ionomers films was measured using quartz crystal microbalance (QCM) (Stanford Research Systems, Sunnyvale, CA). Nafion, LS 1.6, and LS 3.1 films were spin-coated on n-SiO_2_/Au coated 5 MHz crystals followed by the annealing procedure stated in the “Thin-Film Preparation” section. Ionomer film was placed on the crystal holder inside a custom-built plastic humidity chamber connected to an ibidi humidifying system (ibidi USA, Inc., Fitchburg, WI). The total mass of water sorbed by ionomer films (Δ*m*) at each RH was calculated from the corresponding frequency change (Δ*f* ) of the films using the Sauerbrey equation (Equation 4):

(4) $$\begin{array}{l} \Delta f=-\frac{2f_{o}^{2}\Delta m}{A\sqrt{\rho_{q}\mu_{q}}} \end{array}$$

where *f*_*o*_ is the resonant frequency (Hz) of the fundamental mode of the crystal (5 MHz here), *A* is active crystal area (1.27 cm^2^), ρ_*q*_ is the density of quartz crystal (2.648 g/cm^3^), and μ_*q*_ is the shear modulus of quartz (2.947 × 10^11^ g/cm.sec^2^) (Kushner and Hickner, 2017)^1^. The dry mass of each ionomer film was obtained from Δ*f* of the QCM crystal before and after depositing the film (measured at <10% RH). Furthermore, the mass of water uptake for each ionomer film was corrected by subtracting the water uptake of bare QCM crystals at the same %RH since water accumulates within the porous structure of the SiO_2_ layer (Kobayashi et al., 2002; Shim et al., 2015; Kushner and Hickner, 2017). Hydration numbers (λ_*w*_), moles of water per mole of sulfonic acid, at different RH values were calculated using Equation (5):

(5) $$\begin{array}{l} \lambda_{w}=\left(\frac{m_{RH}-m_{0}}{M_{H_{2}O}}\right)\left(\frac{1000}{m_{0}\times IEC}\right) \end{array}$$

where *m*_0_ and *m*_*RH*_ were the sample masses at the dry state and a certain RH, respectively. *M*_*H*_2_*O*_ was the molecular mass of water, and IEC was the ion-exchange capacity of polymer.

#### Density Measurement

The density of the films was measured by dividing the mass/area (from films on n-SiO_2_-coated QCM crystals) by volume/area (from the thickness measurement of a film with a similar thickness on n-SiO_2_ wafers using SE). Here, it was assumed that the mass of the ionomer /area of films made on n-SiO_2_ crystal and n-SiO_2_ wafer were similar.

#### In-plane Reflection Small-Angle X-Ray Scattering (RSAXS) Measurement

Ionic domain characteristics within ionomer films were investigated using the RSAXS technique at ~60% RH using a Rigaku Smartlab Diffractometer operating at 40 kV and 44 mA using a sealed Cu anode X-ray tube with an average wavelength (λ^*^) of X-ray as 1.5418 . RSAXS is a special case of grazing incidence SAXS (GISAXS) technique where the thin film sample is illuminated at grazing incidence angle, but the off-specular reflection signal is captured using a point detector (0D approach). While the 2D detector (used for conventional GISAXS) has the advantage of simultaneously measuring scattering intensities in both in-plane (*q*_*p*_) and out of plane (*q*_*z*_) directions, using the 0D approach, one can resolve one of these (*q*_*p*_ or *q*_*z*_) components at a time (Ogi and Inaba, 2011).

In RSAXS measurement, asymmetric ω/2θ geometry (i.e., ω≠θ, where ω and 2θ are incidence and scattering angles, respectively) was used to collect the data. The scan was performed by intentionally offsetting ω by an appropriate angle, in this case, to avoid the specular reflection (in specular reflection, θ_i_ = θ_f_; where θ_i_ and θ_f_ are the incidence and exit angles, respectively), and captured the off-specular scattering (where θ_i_ ≠ θ_f_) signal (Ito, 2009). The scattered beams were scanned in the angular range (2θ) of 0–6° (step size = 0.02°). From this off-specular scattering, an in-plane scattering component (*q*_*p*_) was obtained, which provided the in-plane structure of the sample (Muller-Buschbaum, 2009). Thin films of Nafion and LS x, with a thickness of ~250 nm, were deposited on n-SiO_2_ wafers. The films were then placed on a sample holder disc and optically aligned. The distance between the sample and the detector was 30 cm. The in-plane scattering vector, *q*_*p*_ is directly related to its scattering angle, 2θ and x-ray wavelength, λ^*^ by Equation (6):

(6) $$\begin{array}{l} q_{P}=\frac{4\pi }{\lambda^{*}}\sin\left(\theta \right) \end{array}$$

The spacing between ionic domains (*d*) within the films was determined based on their primary scattering peak maxima, *q*_*p, max*_ in their specified region (~1.0–2.00 nm^−1^ for LS x; ~1.60–3.50 nm^−1^ for Nafion films) using Equation (7):

(7) $$\begin{array}{l} d=\frac{2\pi }{q_{p,\max}} \end{array}$$

In the cases where the H^+^-form of ionomer did not provide enough scattering contrast (e.g., LS x), the ionomers were converted to Cs^+^-form (Tricoli, 1998; Shi et al., 2016). As mentioned earlier, to prepare an LS x solution, the undissolved fraction had to be removed from the dissolved one (in acetone-water) by centrifugation first. The mass of this undissolved fraction was difficult to quantify but was considerably small and neglected thereby. For ion exchange, the number of moles of H^+^ ions present in 10 wt% LS x solution was thus calculated first [by multiplying the IEC (mmol of –SO_3_H/g polymer) by total weight of LS x ionomer used to make the solution] based on a 10 wt% LS x solution (before centrifugation) and an equivalent number of moles of Cs^+^ ions (in the form of CsCl) was added to the solution subsequently. This yielded a 1 wt% Cs^+^ ion in the final solution. The solutions were spin-coated on prewashed n-SiO_2_ wafers to prepare ~200–230 nm-thick films followed by the annealing procedure described in the “Thin-Film Preparation” section.

#### Atomic Force Microscope (AFM) Imaging

The surface morphology of the annealed Nafion and LS x films on n-SiO_2_ wafers were investigated using AFM (MFP-3D-BIO AFM; Oxford Instruments Asylum Research, Santa Barbara, CA) at ambient condition. An AC240TS silicon cantilever (Olympus Microcantilevers, Tokyo, Japan) with a nominal spring constant of 10.06 N/m and a nominal tip radius of 7 nm was used for imaging in tapping mode. The measurements were performed in 10 × 10 nm^2^ and 1 × 1 μm^2^ scans with a scan rate of 0.2 Hz and 256 scanning lines.

#### Fluorescence Spectroscopic Measurement Using a Rotor Probe

An appropriate volume from a stock solution of CCVJ (in DMSO) was added to LS x solutions to yield 5–10 wt% LS x solutions containing 0.07 wt% CCVJ. n-SiO_2_ wafers were washed following the protocol described in the “Thin-Film Preparation” section. The dye-ionomer solutions were then spin-coated on these prewashed n-SiO_2_ wafers at 3,000 rpm to prepare ~100 and ~250 nm-thick films. The films were annealed following the same procedure mentioned earlier in the “Thin-Film Preparation” section. The films were then placed inside a quartz humidity chamber (2.25″ × 2.25″ × 2.875″) and exposed to air with varied %RH to measure fluorescence spectra using steady-state fluorescence spectroscopy (PTI Quantamaster 400, Horiba, NJ). The excitation wavelength (λ_exc_) of CCVJ was 440 nm, while the emission wavelength (λ_em_) ranged between 470 and 560 nm. Same spectroscopic parameters (excitation/emission slit width = 1 mm; step size = 10 under excitation correction and zero bias) were used to measure fluorescence of all samples.

## Results and Discussions

### Lignin Sulfonate Ionomer (LS X) Synthesis

We first converted the neutral kraft lignin to lignin sulfonate ionomers with different IECs (LS x) following literature (Aro and Fatehi, 2017; Zhang et al., 2017) (please see the Experimental section for synthetic details). The major steps of LS x ionomer synthesis were: (1) a sulfomethylation reaction starting with neutral lignin, (2) acidification to convert –CH_2_SO_3_Na to –CH_2_SO_3_H; and (3) cross-linking the polymer to minimize water solubility (Figures 1B,C). Since this was our first effort to design LS x ionomers, we chose the kraft lignin from Norway Spruce (PDI ~ 2.54) for its commercial availability. All LS x ionomers, reported here were synthesized using the same lot of kraft lignin to ensure the consistent composition of the starting material. Sulfomethylation was done by reacting kraft lignin with Na_2_SO_3_ and HCHO. The use of Na_2_SO_3_ and HCHO together allowed a sulfonation reaction at mild condition, where Na_2_SO_3_ and HCHO reacted together to produce sodium hydroxymethane sulfonate (OH-CH_2_-SO_3_Na) first. This molecule attacked the C-5 positions of benzene rings of kraft lignin in the next step to yield sulfonated lignin in Na-form (–CH_2_SO_3_Na form) (Zhang et al., 2017). Subsequent reaction with HCl converted Na-form to H-form (i.e., –CH_2_SO_3_H form). This sulfomethylation route was more convenient as compared to sulfonation done using Na_2_SO_3_ alone where high temperature and pressure were needed (to attack C-α position of kraft lignin structural units) (Zhang et al., 2017). The Na_2_SO_3_-HCHO based sulfonation strategy was also more advantageous over the one based on butane sultone-NaOH (Tongiani et al., 2005; Rosu et al., 2013; Zhang et al., 2017) as the former one kept the –OH groups intact in the LS x structure (Zhang et al., 2017). These –OH groups can (1) itself take part in ion conduction as hydrogen bond donor and acceptor (Nagamani et al., 2011, 2012), and (2) facilitate hydrophilic ion channel formation alongside the ether linkages and –SO_3_H groups of LS x ionomer chains. The ratio of lignin-to-Na_2_SO_3_ was the key to control the IEC values of LS x ionomers (described in the “Synthesis of Lignin Sulfonate” subsection under the “Methods” section).

The compositions of kraft lignin and LS x ionomers obtained from combustion-based elemental analysis (Table 1) were in close agreement with what was obtained from XPS (Table S1). Both analyses confirmed increased sulfonation of lignin with the increase in IEC. The wt% of S increased from 1.69 wt% (neutral kraft lignin) to 3.48 wt% for LS 1.6 and 7.29 wt% for LS 3.1 as shown in Table 1. Also, XPS spectra provided evidence of the presence of S in the form of –SO_3_H in LS x samples (Figure S1). It is to be noted that the ~1.69 wt% of S present in neutral kraft lignin (Table 1) was majorly due to S-containing functional groups [such as thioethers (–SCH_3_)] other than sulfonic acid (–SO_3_H) and in agreement with prior literature (Upton and Kasko, 2016; Aro and Fatehi, 2017).

**Table 1:** Elemental analysis of kraft lignin and LS x ionomers.

**Ionomers**	**Composition from elemental analysis (wt%)**				
	**C**	**O**	**H**	**S**	**N**
Kraft lignin	67.24	27.44	5.69	1.69	0.50
LS1.6	52.91	31.56	5.28	3.48	<0.50
LS3.1	41.19	38.85	4.43	7.29	<0.50

### Thermal Properties

Due to the complex structure of lignin, its glass transition temperature (*T*_*g*_) is often more difficult to detect than the synthetic polymers. In such cases, the *T*_*g*_ was either represented as a discrete value (Thielemans et al., 2002; Ayoub and Venditti, 2013) or as a range of temperature (Sameni et al., 2014; Thakur et al., 2014). The reported value of *T*_*g*_ for kraft lignin is ~142°C (Thielemans et al., 2002) (or 124–174°C) (Thakur et al., 2014). We found the *T*_*g*_ of LS 1.6 and LS 3.1 to be ~140 and ~170°C, respectively from DSC (Figure S2a). *T*_*g*_ was seen to increase with the increase in IEC of LS x, which was typical and could be attributed to the hindered internal rotation owing to increased intermolecular interactions by hydrogen bonding of –SO_3_H groups (Noshay and Robeson, 1976; Zaidi et al., 2000). Any degradation below 120°C was considered as moisture loss which was prominent for LS 3.1, based on TGA (Figure S2b). A clear degradation started from ~130°C for LS 3.1 and ~150°C for LS 1.6. Within the temperature range of ≥100–400°C, degradation of –SO_3_H groups in many hydrocarbons (Marani et al., 2006; Koziara et al., 2016; Takenaka et al., 2018) and formation of CO, CO_2_, and SO_2_ (Lemes et al., 2010) were reported for sodium lignosulfonate. Thus, the decomposition of LS x above 100°C could be a combined effect of the degradation of macromolecular structure (Hulin et al., 2015) as well the –SO_3_H groups present in LS x ionomers. Since –SO_3_H groups may degrade just above 100°C, we heat-treated (rather than annealed) our LS x samples at 100°C for all our measurements (unless otherwise stated). This was done to ensure that there was no moisture left in LS x films and the dry films can be subjected to gradually increased %RHs.

### Proton Conductivity

**Figure 2:**
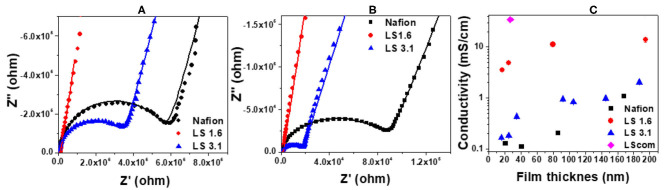
Comparison of electrochemical impedance response of Nafion, LS 1.6, and LS 3.1 films with the thickness **(A)** ~25 nm, and **(B)** ~85 nm. The dotted and straight lines represent the original impedance data and the fits to the data (using an equivalent circuit model), respectively; and **(C)** conductivity values (with error bars) of Nafion, LS 1.6, LS 3.1, and LScom films are plotted as a function of film thickness. All the films were heat-treated at 100°C, and the conductivity values (with fit errors) were measured at ~90% RH and ~22°C.

Figure 2 represents the ion conductivity of ~20–200 nm thick Nafion, LS 1.6, LS 3.1, and LScom films at 90% RH. The impedance curves were fitted into an equivalent circuit model (Figure S3a), and the fits are shown as solid lines in Figures 2A,B. The details on the choice of fitting parameters in the equivalent circuit model (Figure S3a), and the sample fits are shown in Figures S3b, S4. The diameter of the semicircular region of an impedance curve (Figures 2A,B) roughly indicates the resistance of that ionomer film on the gold IDE electrode. A visual inspection of the impedance curves of ~25-nm thick (Figure 2A) and ~85-nm thick (Figure 2B) Nafion, LS 1.6 and LS 3.1 films thus clearly indicated that Nafion has the lowest and LS 1.6 has the highest ion conductivity of all three ionomers at 90% RH for the whole film thickness range studied. The quantitative values of ion conductivity (obtained by fitting the impedance curves in equivalent circuit model), when plotted as a function of film thickness (Figure 2C), showed the trend: LS 1.6 > LS 3.1 ≥ Nafion; where LS 1.6 exhibited proton conductivity about an order of magnitude higher than Nafion over the whole film thickness range. Nafion had an IEC lower than LS 1.6, but even after IEC normalization (i.e., ion conductivity/IEC), the ion conductivity of LS 1.6 (8.12 mS/cm, ~170 nm-thick film at 90% RH) exceeded that of Nafion (1.20 mS/cm, ~170 nm-thick film at 90% RH).

A significant decrease in ion conductivity was observed for LS 1.6 and LS 3.1 when the film thickness went below ~80 nm (Figure 2C) suggesting a stronger confinement effect in films thinner than this value. The confinement effect and interfacial attractive interactions of –OH and –SO_3_H groups of LS x ionomers with the substrate (–SiOH groups of the n-SiO_2_ substrate) may also have been in effect as expected (Dishari and Hickner, 2013). Even at this highly confined state, LS 1.6 retained consistently high proton conductivity (Figure 2C). At a film thickness (~20 nm thick) comparable to the ionomer layer at catalyst interfaces of hydrogen fuel cell electrodes, the conductivity values were ~3.52, ~0.17, and ~0.13 mS/cm for LS 1.6, LS 3.1, and Nafion, respectively (Figure 2C). The data shown above are for films heat-treated at 100°C. When Nafion and LS 1.6 films (~20-nm thick) were annealed at their corresponding *T*_*g*_*s* [100°C (Nafion); 140°C (LS 1.6)], the conductivity values were ~0.13 and ~0.15 mS/cm, respectively (data not shown in Figure 2). All of these suggested that LS x ionomers with controlled IEC can be potential candidates for cost-effective catalyst binders. Commercial lignosulfonate (LScom), on the other hand, showed ion conductivity (~34.72 mS/cm in ~25 nm-thick films, Figure 2C) higher than LS x ionomers at similar film thickness. However, the high conductivity of LScom could be due to its high water solubility and thus limited the practical application of LScom as an ionomer for energy technologies. Despite high IEC, LS 3.1 showed proton conductivity lower than LS 1.6 (Figure 2C). This is not unusual and in some cases it is to excessive water uptake leading to dilution of ions in bulk membranes (Xu et al., 2014; Lin et al., 2017; Li et al., 2018; Long et al., 2019). However, for thin ionomer films, the connection between IEC and ion conductivity may not be that straight forward as many other factors (such as ion mobility, organization of ion-conducting groups/phase segregation, interfacial interactions among water-ionomer and substrate, and more) (Farzin et al., 2019) may come into play in a complex manner to give rise to a certain value of ion conductivity.

### Water Uptake

**Figure 3:**
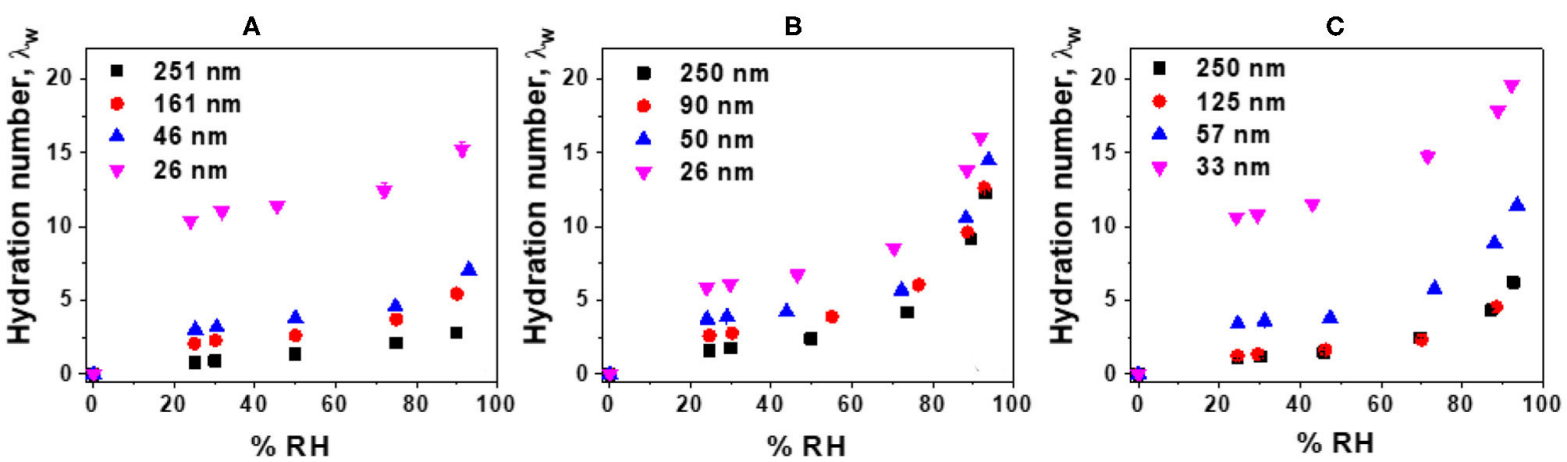
Corrected hydration numbers (λ_*w*_) (with error bars) as a function of %RH: **(A)** Nafion, **(B)** LS 1.6, and **(C)** LS 3.1 films deposited on SiO_2_/Au crystal.

The water uptake of Nafion and LS x films were measured and corresponding hydration numbers (λ_*w*_) were plotted as a function of film thickness and %RH (Figure 3). While Nafion (Figure 3A) and LS 3.1 (Figure 3C) showed thickness-dependent water uptake behavior, the water uptake by LS 1.6 films (Figure 3B) was relatively less sensitive to film thickness. In the thicker films (e.g., ~250 nm-thick films), water uptake was the highest for LS 1.6 with a trend: LS 1.6 (λ_*w*_ ~ 12.22) > LS 3.1 (λ_*w*_ ~ 6.237) > Nafion (λ_*w*_ ~ 2.76). But in thinner films (~25 nm thick), λ_*w*_ of all three ionomers were almost similar [15.38 (Nafion); 16.04 (LS 1.6); 19.59 (LS 3.1) at 90% RH] and very high. The higher water uptake in thinner films has been reported many times (Dishari and Hickner, 2012, 2013; Modestino et al., 2013; Farzin et al., 2019). The consistently high-water uptake of LS 1.6 at all film thickness could be attributed to its relatively low *T*_*g*_ (140°C) and low dry-state stiffness. On the contrary, the high *T*_*g*_ (170°C) and corresponding higher extent of hydrogen bonding between –SO_3_H and –OH groups in the dry polymer may have made LS 3.1 films stiff enough at dry state impeding the water uptake. Having said that, the higher water uptake did not necessarily lead to higher proton conductivity. This observation was consistent with multiple reports on Nafion films with a thickness range similar to ours (Dishari and Hickner, 2012, 2013; Farzin et al., 2019). Despite having similar water uptake, different proton conductivity values of ~25 nm-thick LS 1.6 film (3.6 mS/cm) from LS 3.1 (0.18 mS/cm) and Nafion (0.13 mS/cm) films thus suggested the importance of other factors. Some of the already-identified factors influencing confined ionomeric systems are state (Kim et al., 2003; Seung et al., 2004) (frozen vs. loosely bound vs. free water), mobility (Dishari and Hickner, 2012; Dishari et al., 2018), and distribution (interfacial vs. bulk) (Dishari and Hickner, 2012; Decaluwe et al., 2018; Dishari et al., 2018; Shrivastava et al., 2018) of water, film nanostructure, and ionic domain characteristics (Dishari and Hickner, 2013; Farzin et al., 2019) within the films. The role of water-polymer mobility (or stiffness) and ionic domain characteristics on water uptake and proton conductivity are discussed later in detail with experimental evidence.

Table 2 represents the density of Nafion, LS 1.6, and LS 3.1 at two different thicknesses (25 and 250 nm). LS x ionomer films were less dense than Nafion films at comparable thickness. Lignin macromolecules are branched by nature. Moreover, the acetone-soluble fractions of LS x (used in this work) can possess a higher degree of branching as compared to water soluble fractions (Crestini et al., 2017). This branched architecture may have worked in favor of creating less dense ionomeric materials, unlike other hydrocarbon-based ionomers. For example, a ~250 nm-thick sulfonated polysulfone (S-Radel) film had a density of ~1.45 g/cm^3^ (Dishari et al., 2018), which was higher than LS 1.6 film with a similar thickness (~1.29 g/cm^3^, Table 2). The less compact, branched structure may potentially have facilitated 3D ion channel formation and led to higher ion conductivity of LS 1.6 over Nafion and S-Radel films. On the contrary, LS 3.1 showed lower proton conductivity despite having the lowest film density. While the density was the lowest, the *T*_*g*_ was the highest in LS 3.1 without a significant increase in molecular weight (from LS 1.6), suggesting high intramolecular cross-linking during the chemical reaction. Such chemical cross-linking is likely to involve the –CH_2_OH groups as a result of which the cross-linked material may not be able to leverage the polar –OH groups to create ion conduction pathways.

**Table 2:** Density of Nafion and LS x ionomers in thin films.

**Ionomers**	**IEC**	**Density (g/cc)**	
		**Film thickness (**~**250 nm)**	**Film thickness (**~**25 nm)**
Nafion	0.91	1.89	1.71
LS 1.6	1.6	1.29	1.07
LS 3.1	3.1	1.06	0.92

**Figure 4:**
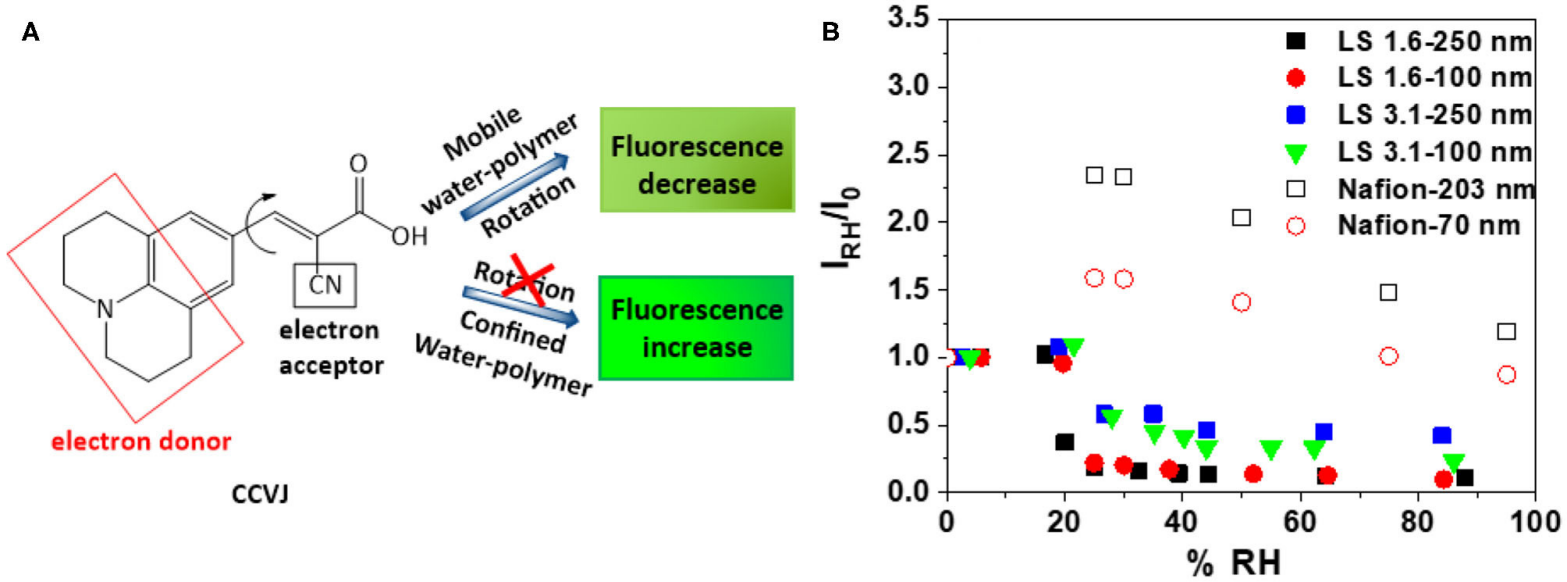
**(A)** Chemical structure and working principle of fluorescent rotor probe CCVJ; and **(B)** relative fluorescence intensity (I_RH_/I_0_) of CCVJ in ~100 nm and ~250 nm-thick LS 1.6, LS 3.1, and Nafion films as a function of %RH [(λ_*exc*_)_CCVJ_ = 440 nm, (λ_*em*_)_CCVJ_ = 470–560 nm].

It was also noted that the thinner films (~25 nm thick) showed lower density than thicker (~250 nm thick) films (Table 2) and was in agreement with prior literature (Shi et al., 2000; Petrina, 2013; Dishari et al., 2018). As compared to a thicker film, a thinner film is made with more dilute (i.e., lower wt% of polymer) and less viscous solution of a polymer with less chain entanglement (Wu et al., 1994). Moreover, we made ionomer solutions with highly volatile solvents [such as acetone (for LS x) or ethanol (for Nafion)]. During the spin-coating of a thinner film, the solvent from the dilute polymer solution evaporates so fast that the polymer chains get locked (non-equilibrium conformation) without getting any chance to reorganize, leaving voids within the thinner films (Shi et al., 2000; Petrina, 2013; Dishari et al., 2018). Chains in such non-equilibrium conformations get an opportunity to equilibrate if the films are annealed above the glass transition temperature (Wu et al., 1994). However, we could not do so for LS x as around *T*_*g*_ (~140–170°C for LS x), the –SO_3_H groups were likely to degrade (discussed earlier). This is why thinner films of LS x ionomers retained lower density than thicker films. Nafion, despite annealing above its *T*_*g*_, showed a decrease in density in thinner films at ambient condition (Table 2). This could be attributed to stronger surface interactions of Nafion with SiO_2_ (an effect similar to polymer chain locking), which retained after annealing as evident from the film stiffening at ~20% RH and beyond (Figure 4, discussed in the next section) and prior works (Dishari and Hickner, 2012, 2013).

### Mobility Prediction

While ionomers can be stiff at dry state, hydration can induce a different degree of stiffening or antiplasticization of ionomer films and increase/decrease the water-polymer mobility within films. Since (1) ion conductivity is a function of both ion concentration and ion mobility; and (2) ion mobility is impacted by water-polymer mobility, it is important to explore the mobility within the ionomer films. A qualitative, but conventional way (Goodelle et al., 2002; Ellison et al., 2004; Dishari and Hickner, 2012, 2013; Dishari et al., 2018) to predict the mobility inside thin ionomer films is to incorporate fluorescent rotor probes inside films and monitor the RH induced changes in its fluorescence response (Figure 4). Here, we used CCVJ (Figure 4A) as the rotor probe. In general, the fluorescence of this dye increases when it experiences a constricted environment (Goodelle et al., 2002; Ellison et al., 2004; Dishari and Hickner, 2012, 2013; Dishari et al., 2018). On the other hand, the fluorescence of the dye decreases when polymeric matrix around it plasticizes or softens. The fluorescence intensity of CCVJ in ionomer films was recorded at dry reference state (*I*_0_) and different elevated RH (*I*_*RH*_). Thus, an increase in the ratio (*I*_*RH*_/*I*_0_) will indicate RH-induced film stiffening or reduced mobility of water molecules and ionomer chains within the film.

As the dry (0% RH) films were hydrated to ~20% RH (Figure 4B), the water-polymer mobility in LS 1.6 and LS 3.1 films changed negligibly (i.e., *I*_*RH*_/*I*_0_ changed negligibly). On the contrary, the Nafion films showed a drastic decrease in mobility (or high antiplasticization) when RH increased from 0 to 20% RH. Above 20% RH, the Nafion films plasticized, but not to the extent like LS x films. The low mobility of water molecules may have contributed to the lower ion conductivity of Nafion films since water molecules have to continuously rotate, break, and form hydrogen bonds for efficient proton conduction.

Another thing to note here is the dry state mobility/stiffness of the films. By normalizing the dry state fluorescence of CCVJ in ionomers films (*I*_0_) with corresponding ionomer film thickness (*L*), we were able to roughly approximate the relative differences in stiffness of the films of the three ionomers at similar thickness. *I*_0_/*L* values for ~250 nm-thick films were 119, 222, and 504 nm^−1^ for Nafion, LS 1.6, and LS 3.1, respectively. This indicated that at dry state, LS 3.1 was already stiffer than LS 1.6 (consistent with *T*_*g*_ of LS 3.1 higher than LS 1.6 shown earlier); while the mobility did not decrease much further upon humidification (Figure 4B). This also indicated that the stiffening of Nafion was more humidity dependent than LS x. Based on *I*_*RH*_/*I*_0_ (Figure 4B) and *I*_0_/*L* values, it can also be inferred that the stiffening of LS 1.6 films (both dry and hydrated state) happened in moderation, favoring its ion conductivity (Figure 2). The higher RH-induced antiplasticization of Nafion (over LS x) could be attributed to the flexible perfluorosulfonic acid chain, which may reach out to the water and –SiOH groups for hydrogen bonding.

### Ionic Domain Characteristics

**Figure 5:**
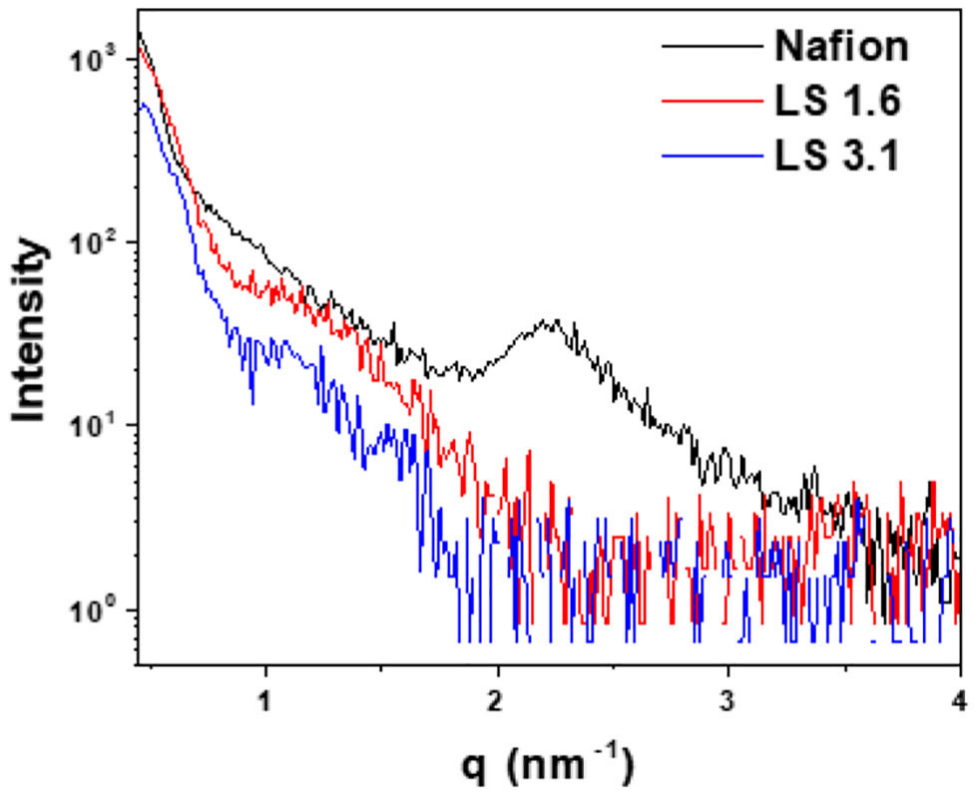
In-plane RSAXS profiles of ~200–250 nm thick Nafion (H^+^ form), LS 1.6 (Cs^+^ form), and LS 3.1 (Cs^+^-form) films on n-SiO_2_ wafers at 60% RH.

Figure 5 represents in-plane RSAXS patterns of Nafion, LS 1.6, and LS 3.1 films at 60% RH. A common practice to (1) enhance the electron density contrast between hydrocarbon matrix and the ionic cluster, and (2) see the ionic domain peaks using RSAXS, is to convert hydrocarbon-based ionomers from H^+^ to Cs^+^ form (Sivashinsky and Tanny, 1983; Li et al., 2010). We did so for LS x ionomers as we could not see any ionic domain peak in LS x films in H^+^ form (LS x-H^+^); while scattering peaks were prominent in Cs^+^ form (LS x-Cs^+^) (Figure 5). On the contrary, Nafion did not show any ionic domain peak in Cs^+^ form in our thin films, while it showed peaks in H^+^ form. A similar observation for Nafion has been reported by others (Tricoli, 1998; Shi et al., 2016). It was suggested that Cs^+^ ions may have strongly interacted with –SO_3_H groups of Nafion causing disruption (Shi et al., 2016) of ionic domains, and the disappearance of ionic domain peak in Nafion-Cs^+^. While it was difficult to be certain whether the evolution of scattering peak in LS x-Cs^+^ was due to (1) increase in electron density contrast between ionic and non-ionic regions of the ionomer, or (2) alter morphologies/internal structure within ionic domains (Shi et al., 2016), we did a couple of measurements. First of all, the UV/Vis absorbance of LS 3.1 (Figure S5a) at 210 and 280 nm [characteristic of aromatic groups in lignosulfonate (Zhou et al., 2016)] did not show any difference between H^+^ and Cs^+^ forms. Second, no significant change in full width at half maxima (FWHM) or shift of ionic domain peak was observed as the extent of Cs^+^ ion exchange was increased (Figure S5b), suggesting an insignificant alteration of ionic domain spacing and distribution in LS 3.1 due to ion exchange. Finally, the ion conductivity (*k*_*f*_) values of LS 1.6-H^+^ and LS 1.6-Cs^+^ were not drastically different [(*k*_*f*_)_LS1.6−H+_ ≈ 2 (*k*_*f*_)_LS1.6−Cs+_]; while Nafion-H^+^ showed conductivity more than an order of magnitude higher than Nafion-Cs^+^ (Shi et al., 2016). Thus, it can be inferred that Cs^+^ ion mainly induced electron density contrast in LS x ionomer films.

Based on FWHM (Table 3), LS 1.6 and LS 3.1 had slightly broader distributions of ionic domain size and spacing as compared to Nafion (~0.74 nm^−1^) in films (Eastman et al., 2012; Kusoglu et al., 2016; Kusoglu and Weber, 2017). The average values of d-spacing (=2π/q_max_) for LS 1.6 (~4.52 nm) and LS 3.1 (~5.61 nm) were larger than Nafion (~2.79 nm) (Table 3). The closed packing of neighboring ionic domains in Nafion films agreed with a large degree of phase mixing as observed in our prior work (Modestino et al., 2013; Farzin et al., 2019). Also, smaller d-spacing was often accompanied by the smaller size of ionic domains for ionomer films (Kreuer, 2001; Hwang et al., 2011; Farzin et al., 2019). The d-spacing we reported here for submicron-thick LS x films were larger than some well-known hydrocarbon-based ionomers even in several tens of micron thick, bulk membrane format (Sivashinsky and Tanny, 1983; Kreuer, 2001; Chang et al., 2013). This possibly suggested that LS x ionomers may have ionic domains bigger than those hydrocarbon and Nafion ionomers. By fitting the ionic domain peak to different models developed for specific self-assembled geometry [e.g., cylindrical, core-shell (spherical), or oblate spheroid (ellipsoid)], ionic domain shape and size are often predicted (Yarusso and Cooper, 1983; Matsuoka et al., 1988; Choi et al., 2010; Kreuer and Portale, 2013; Farzin et al., 2019). We attempted to do so for all three ionomers using Nanosolver (a built-in model of RSAXS system) to obtain the size of ionic domains within films (Table 4).

**Table 3:** q_max_ and domain spacing (d-spacing) of Nafion, LS 1.6, and LS 3.1 films on n-SiO_2_ from in-plane RSAXS data at ~60% RH.

**Ionomers**	**Form**	**Sample thickness (nm)**	**Ionic domain characteristics**		
			**q_**max**_ (nm^**−1**^)**	**FWHM (nm^**−1**^)**	**d-spacing (nm)**
Nafion	H^+^	200 nm	2.25	0.74	2.79
LS 1.6	Cs^+^	250 nm	1.39	0.92	4.52
LS 3.1	Cs^+^	250 nm	1.12	0.85	5.61

**Table 4:** Comparison of the fitting parameters of ~200–250 nm-thick Nafion, LS 1.6, and LS 3.1 films at 60% RH obtained by fitting ionic domain peaks (RSAXS) using Nanosolver^*^.

**Model**	**Parameters**	**Nafion**	**LS 1.6**	**LS 3.1**
Core-shell (spherical)	Average diameter (2r) (nm)	1.85 ± 0.05	10.50 ± 0.20	8.95 ± 0.25
Oblate spheroid (ellipsoid)	Average diameter (2r) (nm)	1.55 ± 0.05	7.50 ± 0.20	6.00 ± 0.20
	Aspect ratio (a)	1.00	5.50	4.50
	Length of long axis (2ar) (nm)	1.55	41.25	27.00
Cylindrical	Average diameter (2r) (nm)	-	5.00 ± 0.20	4.50 ± 0.20
	Aspect ratio (a)	-	3.50	3.00
	Length (2ar) (nm)	-	17.50	13.50

*The dimensions for each ionic domain geometry are schematically shown in Figure S6a. R-value, a measure of the quality of the fit of ionic domain peaks, was found to be low (R ~ 0.06–0.22). The variations (±) in average diameter mean the values within this range fits the RSAXS curves equally well.

For Nafion, we used a core-shell-based spherical model only as of the existing literature on Nafion bulk membrane (Gebel, 2000; Haubold et al., 2001) and thin films (Farzin et al., 2019) strongly support that. At 90% RH, the volume fraction of water sorbed by Nafion thin film was <0.25, which did not support the formation of cylindrical, connected morphology (Farzin et al., 2019). Even when we tried to fit ionic domain peaks of Nafion films in a spheroid model (Table 4), we found an aspect ratio of 1.0, which strongly supported the formation of spherical ionic domains in Nafion films.

On the other hand, above critical aggregation concentration (CAC), a broad range of geometries of self-assembled structures has been proposed for water-soluble sodium lignosulfonate [CAC ~ 0.38 g/L (Qiu et al., 2010)], such as oblate (in solutions and films) (Pasquini et al., 2002; Vainio et al., 2008; Yan et al., 2010), irregularly shaped flat disc-like (in films) (Goring et al., 1979; Qiu et al., 2010), and spherical (in solution) (Kontturi, 1988; Qiu et al., 2010). Please note that not all of this water-soluble lignosulfonate is commercial (i.e., some of those were made from different natural sources in the lab). Therefore, we avoided using the term “LScom” onward, whenever appropriate. Also, we should keep in mind that all the literature reporting varied morphology do also vary in the sources of lignin (Goring et al., 1979; Pasquini et al., 2002; Vainio et al., 2004), the process of making lignosulfonates (Rezanowich et al., 1964; Goring et al., 1979; Yan et al., 2010), molecular weight and polydispersity of polymers, and solution/film preparation conditions (Kontturi, 1988; Vainio et al., 2008); and no straightforward trend was found in structural geometry as a function of any of the above-mentioned parameters. This suggested that we should evaluate different possible geometries of LS x particles/domains in films rationally. We, therefore, primarily selected three major ionic domain geometries to fit the ionic domain peaks in LS 1.6 and LS 3.1 films for subsequent evaluation: core-shell, spheroid, and cylindrical (disc-like) (as shown in Figure S6a). The corresponding best-fit parameters for all three ionomers are tabulated (Table 4). Representative fits of the ionic domain peaks using Nanosolver are shown in Figures S6b,c.

By fitting the ionic domain peaks into a core-shell model, the average size of ionic domains was found to be the highest for LS 1.6 (~10.5 nm) and the lowest for Nafion (~1.85 nm) (Table 4). The size of the ionic domain as big as ~9–10.5 nm appeared to be too high [the largest reported ionic domain is ~4 nm of ~25–50 μm-thick Nafion membrane at hydrated state (Gebel, 2000)]. However, the diameter of a single lignosulfonate macromolecule was often roughly approximated to be ~8 nm (based on particle size distribution from dynamic light scattering measurements in solution) (Yan et al., 2010). This suggested that if our LS x films truly have core-shell ionic domain structure, the individual domains are likely to be made of single LS x macromolecules where the core is the ion-conducting region.

While the spherical morphology of lignosulfonate particles in solution is reported, the spherical morphology at the interface (Qiu et al., 2010) or in concentrated solution (Kontturi, 1988; Vainio et al., 2008) of these molecules is debatable (based on the interpretation of the exponent value in Mark-Howink equation correlating diffusion coefficient of a macromolecule to its molar mass) (Vainio et al., 2008). Our AFM topographical (Figure S7) and 3D (Figure S8) images of LS x ionomer films clearly indicated large oblate spheroid-like, elongated, aggregated structures standing perpendicular to the substrate. Therefore, we looked for more evidences based on SAXS to comment on the ionic domain size within LS x films. Vainio et al. (2008) fitted SAXS data of lignosulfonate particles, which yielded oblate-shaped particles with an aspect ratio of 3.5. The presence of such oblate spheroid structures has also been evidenced in solid samples of dry kraft lignin (SAXS, USAXS) (Vainio et al., 2004), Langmuir-Blodgett films of saccharified lignin (AFM) (Pasquini et al., 2002), and drop-cast lignosulfonate films on silicon flakes [environmental scanning electron microscopy (SEM)] (Yan et al., 2010). All of these prior evidences convinced us that the oblate spheroid (or ellipsoid) model can be a rational choice to fit our RSAXS data and obtain ionic domain size on LS x thin films.

The ionic domain size trend (LS1.6 > LS 3.1 > Nafion) was still valid when the dimensions of ionic domains of Nafion using the core-shell model and LS x using the ellipsoid model were compared (Table 4). When we employed the ellipsoid (or oblate spheroid) model to fit RSAXS data of our LS x films, the following dimensions of ionic domains were obtained: LS 1.6: diameter (2r) ~ 7.5 nm; length (2ar) ~ 41.25 nm; LS3.1: diameter (2r) ~ 6 nm; length (2ar) ~ 27 nm. The aspect ratios were found to be 5.5 (LS 1.6) and 4.5 (LS 3.1), which were higher than that for lignosulfonate in solution (~3.5) (Vainio et al., 2008). This could be attributed to the conformational difference between solution (Vainio et al., 2008) and solid-state as well as the nature of the solvent used [LS x (acetone-water) and lignosulfonate (water) used (Vainio et al., 2012)]. All of these factors can impact the solution and solid-state self-assembly and so are the aspect ratios of ionic domains.

If the average dimension of single LS x macromolecules in films was closer to what was reported for single lignosulfonate macromolecules in solution (~8 nm) (Yan et al., 2010), the oblate spheroidal domains in LS x films from RSAXS [length ~ 41 nm (LS 1.6); ~27 nm (LS 3.1); Table 4] suggested the likelihood of aggregation or association of multiple LS x macromolecules to form single oblate spheroidal ionic cluster or particle. Such association of up to 2–10 oblate spheroid-shaped lignosulfonate macromolecules has been suggested in solution (SAXS) (Vainio et al., 2008, 2012) and in the solid-state (SEM) (Yan et al., 2010). However, how the LS x molecules are organizing within these oblate clusters requires further investigation to understand. Based on the common understanding of ionomers in solid films, it can be predicted that when the %RH is not extremely high (i.e., when RH ≤ 90%), the sulfonic acid groups of LS x are located at the interior, and aromatic rings are located at the surface of the macromolecules. Thus, the hydrophobic interaction (Deng et al., 2012; Vainio et al., 2012; Ma et al., 2018) may drive the intermolecular association to give large aggregates. The AFM topography (Figure S7) and 3D images of LS x films (Figure S8) suggested a much stronger aggregation and formation of larger elongated clusters (as compared to the cluster size obtained from RSAXS, Table 4) at the air/film interface. However, as the film thickness decreased to ~20–25 nm, these ellipsoidal perpendicular features disappeared from AFM images (Figure S9). Despite that, the high ion conductivity of ~20 nm-thick LS 1.6 films (Figure 2C) suggested a rearrangement/reorientation of ionic domains still favorable for proton conduction.

On another note, the possibility of having disc-like/cylindrical structures [proposed by others (Goring et al., 1979; Vainio et al., 2012) of lignosulfonate at the interface] is less in LS x films. This is based on a simple hypothesis: if multiple cylinders are stacked to form large-scale structures, it is highly unlikely to obtain such elongated oblate shapes (seen in AFM images, Figure S8) out of cylindrical units, unless cylinders with gradually smaller diameter are systematically stacked from the center to edges. Without applying some precise self-assembly-based film preparation techniques, such hierarchical stacking may not be possible. With limited prior information available about lignin sulfonate similar to what we designed, it may not be ideal to try to make comments with more certainty than this.

### Why Did LS x Exhibit High Thin-Film Ion Conductivity?

High acidity of ion-conducting groups (Chang et al., 2011), and connected, hydrogen-bonded network of water molecules (Ueki and Watanabe, 2008) with sufficient rotational mobility (Laage, 2006) are considered as prerequisites for efficient proton conduction. As opposed to the long, flexible perfluorosulfonic acid side chains of Nafion (pK_a_ ~ −14), the pK_a_ of LS x should be less negative with short methylsulfonic acid side chains (predicted based on pK_a_ reported for –C_6_H_4_SO_3_H as −2.5) (Chang et al., 2011). Therefore, LS x with low acidity was expected to exhibit lower proton conductivity as compared to highly acidic Nafion (Chang et al., 2011). However, it appeared that the significantly larger ionic domains/clusters (comprised of multiple LS x macromolecules) (Table 4) helped LS x films overcome the side-chain acidity effect. The branched, 3D, less-compact (low density, Table 2) structures likely offered ample free spaces (or free volumes) within the hydrophilic interior of individual LS x macromolecules. Based on experimental evidences, we anticipated that within this hydrophilic interior, the close proximity of –SO_3_H and polar ether and –OH groups attracted water molecules, allowed them to move, and assisted connectivity between individual macromolecules to create ion-conducting cluster networks. The rotor probe experiments (Figure 4) provided evidence of high-water mobility within these networks; while the high aspect ratio of the LS 1.6 clusters (based on the spheroid model) supported the good connectivity among clusters made of individual LS x macromolecules. Together, it created a bulk-like ion-conducting environment [which so far has been defined as ~4-nm size ionic domains in bulk Nafion membrane (Gebel, 2000)] in submicron-thick LS x films. As the film thickness of LS 1.6 approached ~20 nm, the protruded, ellipsoidal, perpendicular features disappeared (Figure S9), but the ion conductivity was still maintained at high value suggesting reorientation/alignment of ionic domains, which was still favorable for ion conduction. As opposed to some of the traditional hydrocarbon-based ionomers (Kreuer, 2001; Seung et al., 2004; Peron et al., 2011; Chang et al., 2013), the phase segregated, large, and connected ionic domains showed the promise of LS 1.6 as an ionomer for thin ion-conducting materials.

LS 3.1, on the other hand, showed ion conductivity lower than LS 1.6 but higher than Nafion in most of the thickness range we studied (Figure 2C). The low ion conductivity of LS 3.1 was in agreement with its higher dry-state *T*_*g*_ (~170°C) as compared to LS 1.6 (*T*_*g*_ ~ 140°C) and could be attributed to the higher extent of hydrogen-bonding between –SO_3_H and –OH groups in LS 3.1 As a result, LS 3.1 films were already stiff enough at the dry state (further supported by *I*_0_/*L* of CCVJ) and might have impeded the water uptake (Figure 3C). At hydrated state, LS 3.1 films retained this high stiffness (insignificant change in *I*_*RH*_/*I*_0_, Figure 4) and low water mobility, which had a negative impact on proton conductivity. On the other hand, both Nafion and LS 1.6 films were less stiff than LS 3.1 films in the dry state. But upon humidification, Nafion films significantly stiffened up, while LS 1.6 films did not. The comparison of *I*_*RH*_/*I*_0_ (Figure 4) values suggested that at 60% RH, Nafion was still approximately six times stiffer than LS 1.6 and LS 3.1. Therefore, the low stiffness, high water mobility, larger ionic domains all together led to such a high ion conductivity of LS 1.6 as compared to Nafion and LS 3.1 films.

## Conclusions

We have systematically synthesized sulfonated kraft lignin (LS x) (from Norway Spruce) with controlled IECs and limited water solubility and explored the potential of LS x as an ion conductor in submicron-thick films. While commercial lignosulfonate (LScom) is completely water soluble, we successfully limited the water solubility of LS x (through adjustable polymer cross-linking), while making them suitable for hydration-mediated proton conduction. The ion conductivity followed a trend: LS 1.6 > LS 3.1 ≥ Nafion in 20–200 nm-thick films at 90% RH. Using LS 1.6, ion conductivity about an order of magnitude higher than Nafion was achieved in submicron-thick films, which suggested the great potential of LS x-based ionomers in overcoming ion conduction limitations at the ionomer-catalyst interface. Water uptake, density, dimension and d-spacing of ionic clusters, and qualitative estimation of the extent of water-polymer mobility (or film stiffness) were obtained for all three ionomers in films. The higher value of proton conductivity of LS 1.6 was believed to be an individual or synergistic effect of: (1) the presence of –OH groups in close proximity of –SO_3_H groups in highly branched, 3D structure; (2) low film density, (3) higher water uptake (in thicker films only), (4) lower stiffness (higher water mobility), and (5) significantly larger ionic domains with domain connectivity. On the other hand, denser Nafion films with lower water uptake (thicker films only), smaller ionic domains, and significant hydration-induced antiplasticization led to lower ion conductivity of Nafion films over LS 1.6 films (and LS 3.1 over some thickness range). For LS 3.1 films, higher dry-state *T*_*g*_ and stiffness were observed. This suggested that higher IEC of an ionomer may not be beneficial to ion conductivity if the ionomer chains and water experience stronger confinement. As LS 1.6 ionomer, made from cheap and abundant kraft lignin, exhibited much higher ion conductivity over Nafion in films with a thickness comparable to catalyst binder layer (~2–30 nm), it may have a great potential to address ion conduction limitations. Such understanding of ion conduction behavior of LS x in thin films, along with further consideration of material viability (to be reported in the future) can inform and guide the future design of lignin-based ionomers for energy conversion and storage devices. This also potentially indicates a new way to valorize waste lignin from industrial (biorefinery or pulp/paper industries) and natural resources.

## Data Availability Statement

The raw data supporting the conclusions of this article will be made available by the authors, without undue reservation.

## Author Contributions

SF performed sulfonation, water uptake, TGA, DSC, RSAXS, SE, XPS, elemental analysis, density, CCVJ experiments, and fitting of ionic domain peak in RSAXS. TJ performed EIS measurements, and EZ performed the UV/Vis experiments, while TJ and EZ worked together for AFM experiments. SC determined molecular weight and helped SF with the sulfonation of kraft lignin. SD, SF, and TJ were involved in manuscript writing. SD proposed the main concept of the work, supervised the experimental plans, led the manuscript writing, and revisions. All authors contributed to the article and approved the submitted version.

## Conflict of Interest

The authors declare that the research was conducted in the absence of any commercial or financial relationships that could be construed as a potential conflict of interest.

## Footnotes

^1^Operation and Service Manual-Research Quartz Crystal Microbalance- Inficon.
