# Supplementary material for: Ionomers From Kraft Lignin for Renewable Energy Applications
Source: Front Chem. 2020 Aug 26;8:690. doi: 10.3389/fchem.2020.00690 (PMC7480228; doi:10.3389/fchem.2020.00690)
Supplement: Supplementary file 1 [file Data_Sheet_1.pdf]

Supporting Information

Ionomers from Kraft Lignin for Renewable Energy Applications

Seefat Farzin,[1] Tyler J. Johnson,[1] Shyambo Chatterjee,[1] Ehsan Zamani,[1] and Shudipto K. Dishari[1,]*

[1] Department of Chemical and Biomolecular Engineering, University of Nebraska-Lincoln, Lincoln, Nebraska 68588, United States

*Corresponding author's email: sdishari2@unl.edu

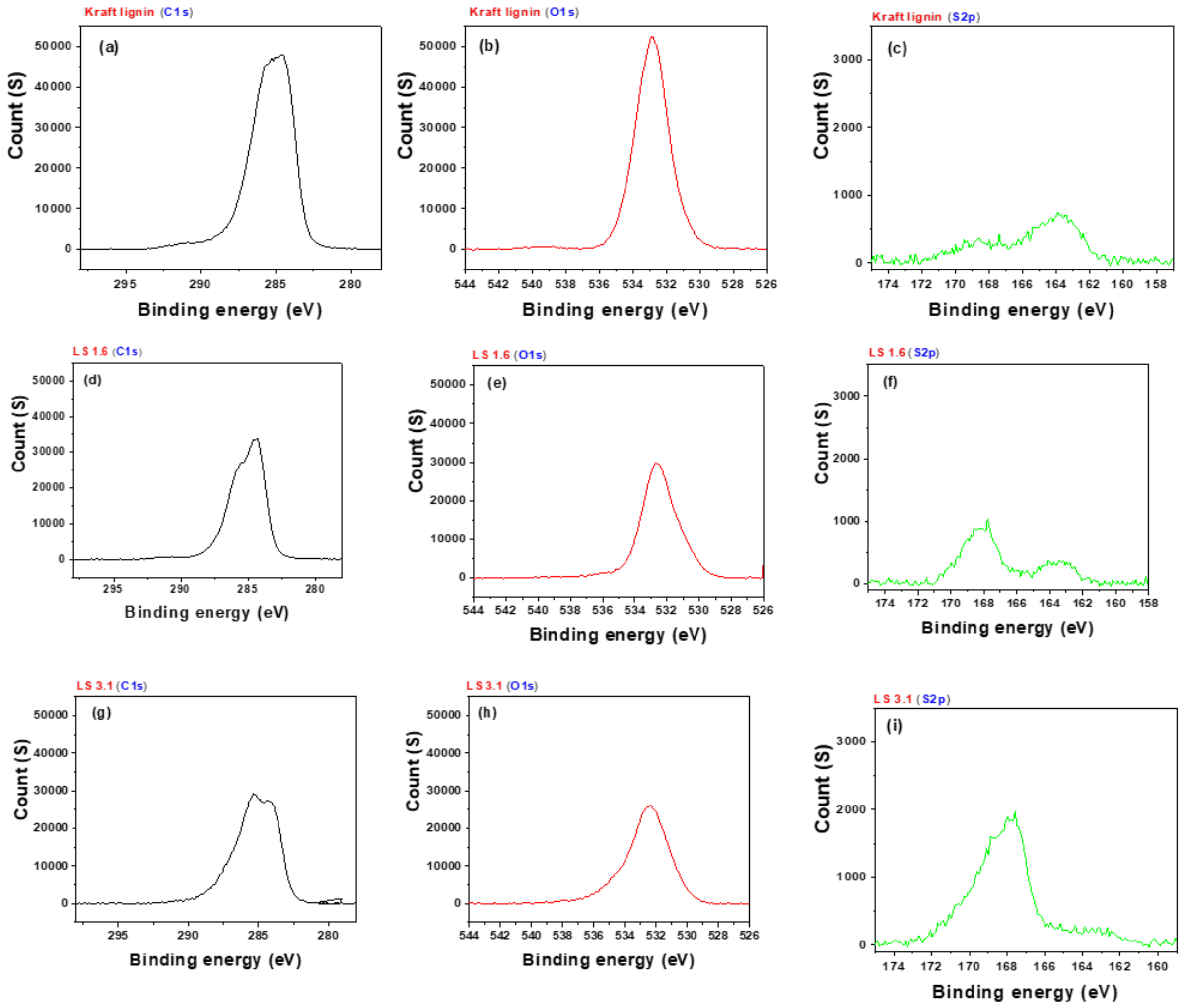

Figure S1: XPS spectra (C1s (left); O1s (mid); S2p (right)) of kraft lignin (a,b,c), LS 1.6 (d, e, f), and LS 3.1 (g, h, i).

XPS spectra. X-ray photoelectron spectroscopy (XPS) with a beam of X-ray (Al-Kα with the energy of 1486.6 eV) was performed at Nebraska Center for Materials and Nanoscience (NCMN) for elemental analysis (wt% of carbon (C), oxygen (O) and sulfur (S)) of the neutral kraft lignin and ionomer samples. For XPS measurements, the vacuum was kept at 9.0×10^{-8} Torr. The C1s, O1s, and S2p spectra for all the samples are shown in the supplementary information (Figure S1). Each component analysis was performed twice. The elemental compositions were quantified using manufacturer's peak-fitting software and are also shown in Table S1.

Table S1: Elemental analysis of kraft lignin, and LS x ionomers.

Ionomers	***Composition from XPS (wt%)***		
	C	***O***	***S***
Kraft Lignin	*68.20*	*29.74*	*2.05*
LS1.6	*67.06*	*29.75*	*3.19*
LS3.1	*57.60*	*35.32*	*7.08*

A gradual increase in the intensity of ~ 169 eV peak (correspond to S from –SO_3H groups(Upare et al., 2013)) with the increase in IEC of LS x samples provided evidence of the increased degree of sulfonation with IEC (Figure S1c, f, i, l). The corroborates with the quantitative values wt% S presented in table S1and the combustion-based elemental analysis (Table 1).

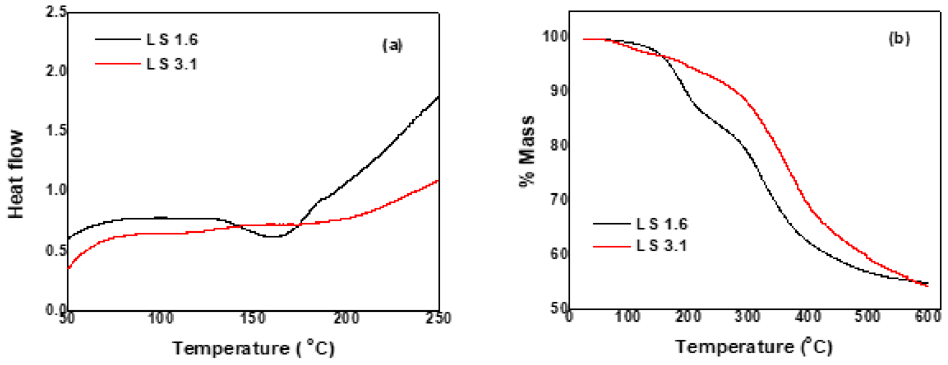

Figure S2: (a) T_g analysis of LS 1.6 and LS 3.1 samples using differential scanning calorimetry (DSC); (b) % Weight loss of LS 1.6 and LS 3.1 ionomers measured *via* thermogravimetric analysis (TGA).

EIS measurements and fits of impedance spectra. In modeling of impedance data of an electrochemical system using an equivalent circuit model, it is important to carefully construct the model in the purview of the processes occurring within the system. With this in mind, we used the model shown in Figure S3 to

describe our ionomer thin film-based electrochemical system. The model consists of several circuit elements. The series resistance R_s account for intrinsic resistance of the measurement system such as the resistance of the connecting cables and the probes. The series inductor L_1 accounts for the inductance of the connecting cables which is typically important for frequencies greater than 1 MHz only. A typical measurement of a Nafion thin film fitted to the equivalent circuit model is shown in Figure S3b. Notable features of the response include a flattened semicircle at high frequencies and a slightly off-vertical line at low frequencies. To describe the response at high frequencies, a parallel combination of constant phase element (CPE_f) and resistor (R_f) was used. In the equivalent circuit (Figure S3). The resistor R_f represents the ionomer film resistance, while the constant phase element CPE_f represents the imperfect capacitor-like behavior of the ionomer film. The constant phase element was used instead of an ideal capacitor to account for inhomogeneity and surface roughness between the parallel planes of the capacitive surfaces. (Yadav and Fedkiw, 2012; Paul et al., 2014) In contrast, a parallel combination of a resistor and ideal capacitor would produce a semicircle with a definite radius. (Paul et al., 2014) The parallel combination of R_f and CPE_f manifests the flattened semicircular portion of the response. (Paul et al., 2014) Finally, the constant phase element CPE_{dl} in series with R_s (Figure S3a) represents the double-layer capacitance at the interface of the ionomer and the IDA electrodes. While an ideal capacitor at low frequencies would produce a vertical line, there is a notable positive slope in the observed response, so the constant phase element was chosen.

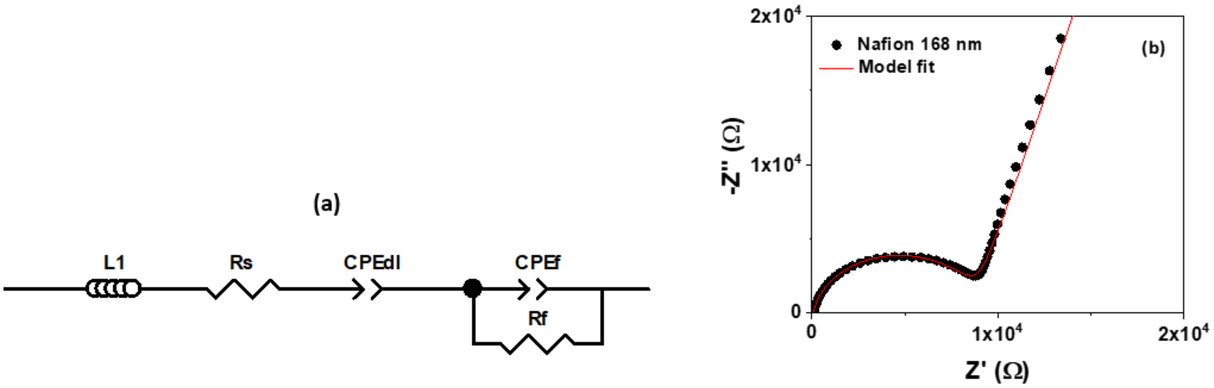

Figure S3: (a) Equivalent circuit model used to fit the EIS data for the ionomer/IDA electrochemical system; (b) typical impedance response of Nafion thin film (thickness ~ 168 nm) fitted to this equivalent circuit model. The flattened semicircle at high frequencies is represented by the parallel combination of R_f and CPE_f, while the slightly off-vertical line at low frequencies is represented by CPE_{dl}.

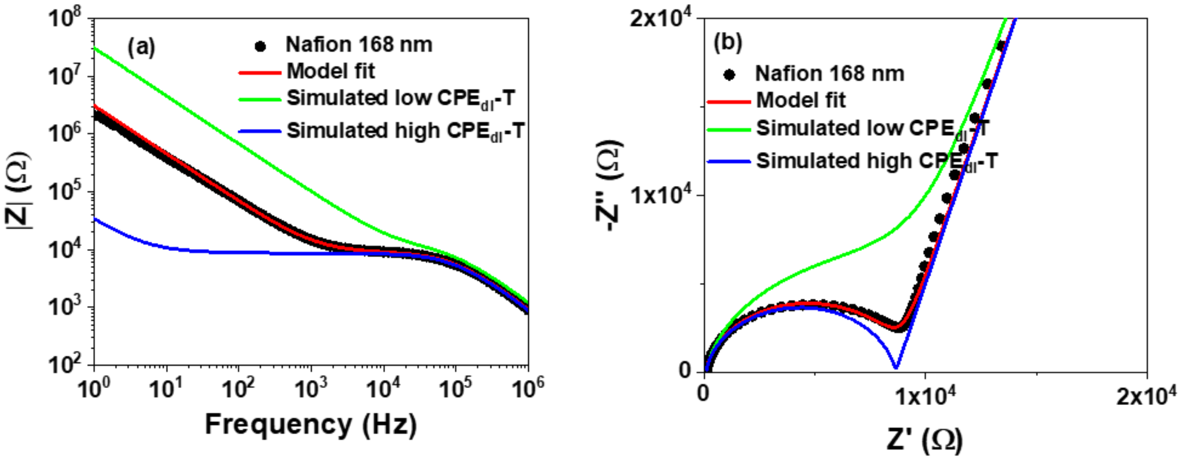

Figure S4: Equivalent circuit fit (a) Bode plot; (b) Nyquist plot of a 168 nm thick Nafion film at 90% RH.

The goal for modeling the impedance data in this work was to quantify the film resistance, R_f, so that the film conductivity, k_f, could be calculated by using equation (3). In this work, d = 100 um, l = 8 mm, and N = 110. To further investigate the shape of the observed impedance response and its relation to the equivalent circuit model, we fitted the data for a 168 nm thick Nafion film and varied the value of CPE$_{dl}$-T (The symbol "T" represents the value of the CPE$_{dl}$). The resulting impedance magnitude and complex impedance plots are shown in Figure S5a and b, respectively, and the fitting parameters for the model fit are shown in Table S2. In the model fit, the value of CPE$_{dl}$-T was 6.98×10^{-8} F. When the value of CPE$_{dl}$-T was decreased by one order of magnitude, the semicircle became less sharp (Figure S5b), and the plateau in Figure S5a was shortened significantly. However, when the value of CPE$_{dl}$-T was increased by two orders of magnitude, a semicircle with a sharp distinction was observed in Figure S5b, and the plateau in Figure S5a was extended to lower frequencies. The difference in observed responses is explained by the difference in the value of CPE$_{dl}$-T relative to the value of CPE$_f$-T. We found that the difference between these values should be approximately two orders of magnitude to achieve a semicircular-type response for this system, which was also supported by the literature (Paul et al., 2014).

Table S2: Summary of equivalent circuit modeling parameters for the 168 nm thick Nafion film. *

Parameters	***Values***
L_1	1.17×10^{-5} *H*
R_s	*34.51 Ω*
CPE_{dl}*-T*	6.98×10^{-8} *F*
CPE_{dl}*-P*	*0.83*
CPE_f*-T*	8.12×10^{-10} *F*
CPE_f*-P*	*0.90*
R_f	*8598 Ω*

* The symbol "T" and "P" indicates the magnitude and power (e.g. P = 1 is an ideal capacitor) of CPE_{dl}. The same is applicable for CPE_f.

UV/Vis absorbance measurements: 1 wt% of dry LS 3.1 was dispersed in water and then centrifuged at 8500 rpm for 2 min to remove the undissolved fraction. The supernatant was collected and diluted in water to measure UV/Vis absorbance using Perking-Elmer Lambda 40 UV/Vis spectrophotometer. The moles of H^+ were calculated based on the IEC and total weight of LS 3.1. The same number of moles of Cs^+ was added to 1wt% LS3.1 solution and the absorbance was measured subsequently (Figure S7a).

For thin film preparation with Cs^+ ion, a similar calculation was performed and mixed the Cs^+ to 10wt% LS 3.1 solutions to yield 0.75, 1, and 1.15 wt% of the required amount of Cs^+ ions separately. The solutions were then spin-coated on prewashed silicon wafers to make ~200 nm thick LS 3.1 films. The RSAXS patterns of these LS 3.1 films with 0.75, 1, and 1.15 wt% Cs^+ are shown in Figure S8b.

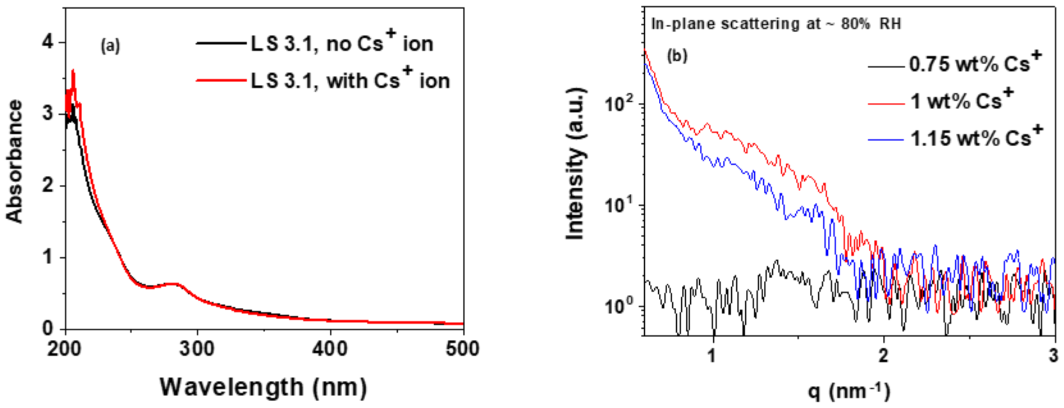

Figure S5: (a) UV/Vis absorbance of LS 3.1 in the presence and absence of Cs^+ ion in the solution; (b) RSAXS pattern of ~200 nm thick LS 3.1 film in the presence of a different concentration of Cs^+ in the spinning solution.

Ionic domain peak fitting (RSAXS).

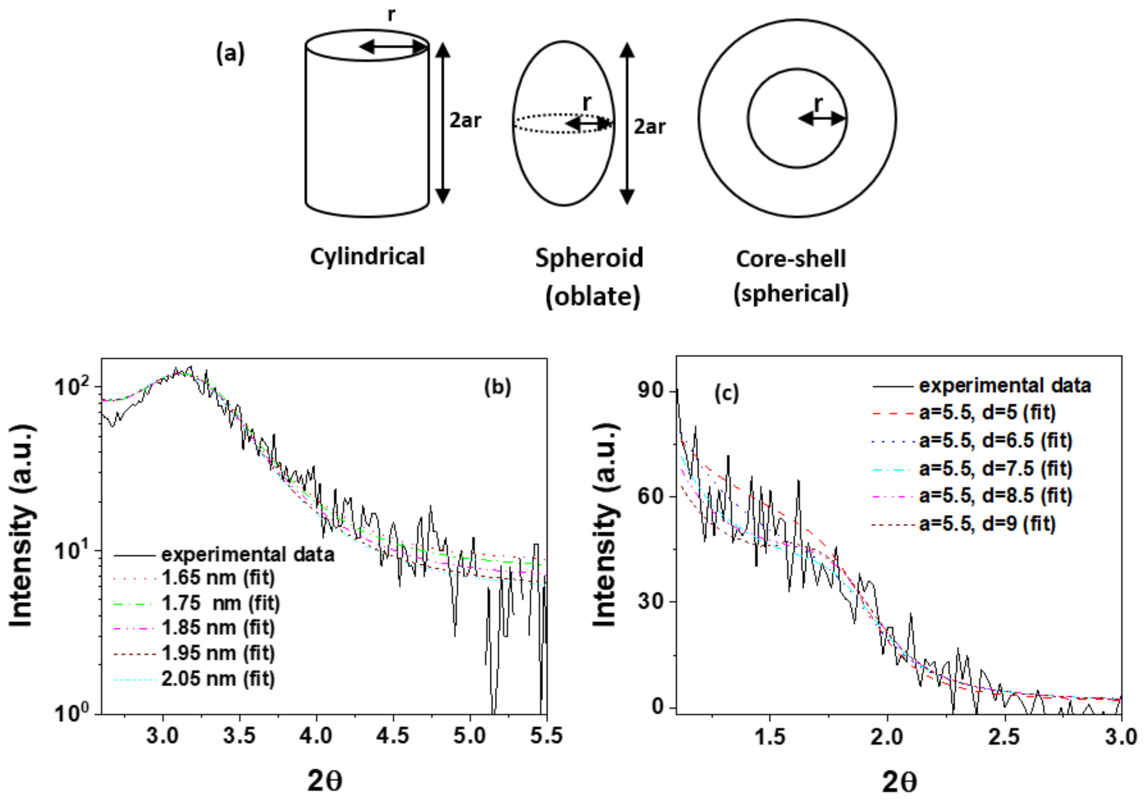

Figure S6: (a) Geometry of particle-specific shapes used to fit RSAXS data; fitting of RSAXS ionic domain peak of (b) ~251 nm thick Nafion film using core-shell model, and (c) ~230 nm thick LS 1.6 film using the spheroid model in Nanosolver for different diameters of the core at 60%RH.

AFM images.

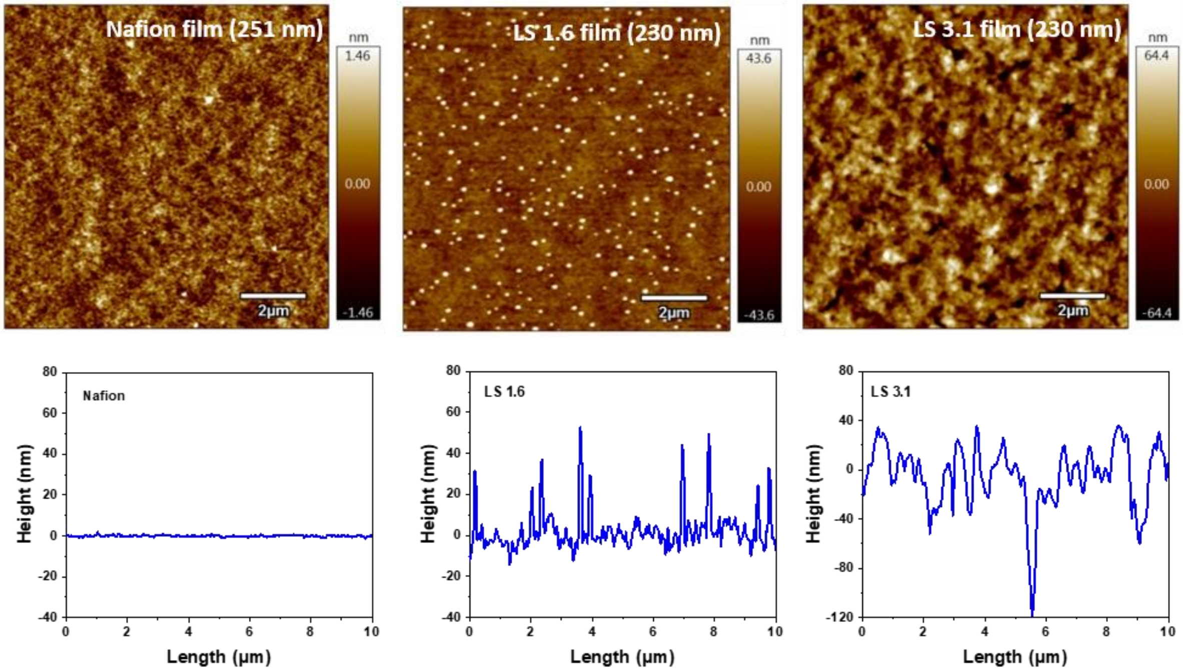

Figure S7: Tapping-mode topographical (top row) AFM images of LS 1.6 (left), LS 3.1 (middle), and Nafion (right) films with thickness ~230 nm. The corresponding height profiles are shown in the bottom row.

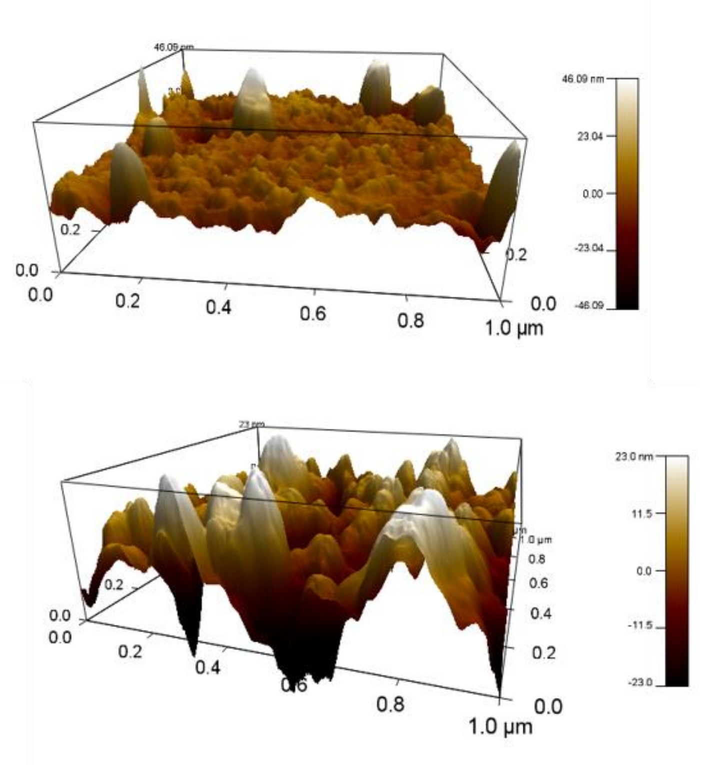

Figure S8: Tapping-mode 3-dimensional AFM images of LS 1.6 (top) and LS 3.1 (bottom) films with thickness ~200 nm.

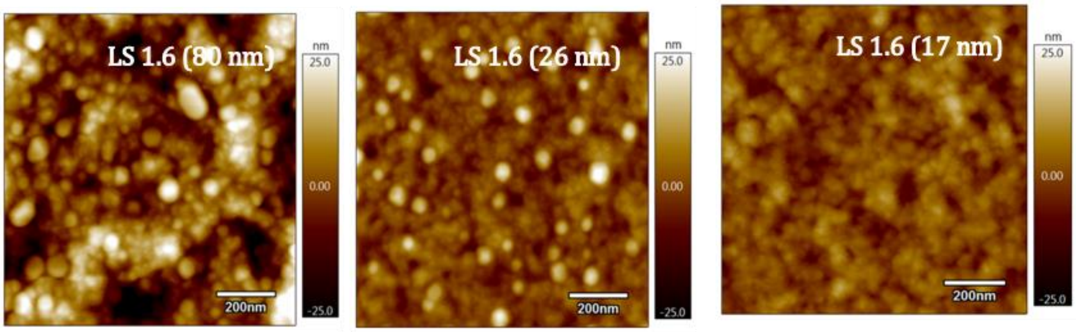

Figure S9: Tapping-mode topographical AFM images of LS 1.6 films with thickness 80 nm, 26 nm, and 17 nm (from left to right).

References:

Paul, D. K., McCreery, R., and Karan, K. (2014). Proton transport property in supported Nafion nanothin films by electrochemical impedance spectroscopy. *J. Electrochem. Soc.* 161, F1395–F1402. doi:10.1149/2.0571414jes.

Upare, P. P., Yoon, J., Kim, M. Y., Kang, H., Hwang, D. W., Hwang, Y. K., et al. (2013). Chemical conversion of biomass-derived hexose sugars to levulinic acid over sulfonic acid-functionalized graphene oxide catalysts. *Green Chem.* 15, 2935–2943. doi:10.1039/c3gc40353j.

Yadav, R., and Fedkiw, P. S. (2012). Analysis of EIS technique and Nafion 117 conductivity as a function of temperature and relative humidity. *J. Electrochem. Soc.* 159, B340–B346. doi:10.1149/2.104203jes.
